# A megastudy on the predictability of personal information from facial images: Disentangling demographic and non-demographic signals

**DOI:** 10.1038/s41598-023-42054-9

**Published:** 2023-11-29

**Authors:** Yegor Tkachenko, Kamel Jedidi

**Affiliations:** Marketing Department, Columbia Business School, New York, 10027 USA

**Keywords:** Information technology, Computational science, Statistics

## Abstract

While prior research has shown that facial images signal personal information, publications in this field tend to assess the predictability of a single variable or a small set of variables at a time, which is problematic. Reported prediction quality is hard to compare and generalize across studies due to different study conditions. Another issue is selection bias: researchers may choose to study variables intuitively expected to be predictable and underreport unpredictable variables (the ‘file drawer’ problem). Policy makers thus have an incomplete picture for a risk-benefit analysis of facial analysis technology. To address these limitations, we perform a megastudy—a survey-based study that reports the predictability of numerous personal attributes (349 binary variables) from 2646 distinct facial images of 969 individuals. Using deep learning, we find 82/349 personal attributes (23%) are predictable better than random from facial image pixels. Adding facial images substantially boosts prediction quality versus demographics-only benchmark model. Our unexpected finding of strong predictability of iPhone versus Galaxy preference variable shows how testing many hypotheses simultaneously can facilitate knowledge discovery. Our proposed L1-regularized image decomposition method and other techniques point to smartphone camera artifacts, BMI, skin properties, and facial hair as top candidate non-demographic signals in facial images.

## Introduction

Two distinct modern AI technologies can be applied to human facial images: facial recognition and facial analysis. Facial recognition involves matching a given photo to the one observed in the past, and then to a unique identifier for the photo’s owner, subsequently retrieving known information about the individual. Facial analysis involves predicting individual data using statistical inference from the image itself and can be performed even on individuals one sees for the first time. The focus of this paper is on facial analysis. The cumulative evidence from academic research clearly shows that facial analysis, based on machine learning or direct human examination, can be used to predict different types of personal data directly from facial images of people, including age, gender, race^1^, personality^2^, names^3^, social class^4^, political orientation^5^, propensity for aggressive behavior^6^, and homosexuality^7,8^. Reported accuracy rates vary but can reach relatively high levels (e.g., 0.7–0.73 range for liberal vs. conservative classification^5^; 0.81 for male homosexuality^7^; >0.95 for gender and race classification^1^). Admittedly, the exact causal mechanism that enables predictions from facial images can be controversial. For instance, in case of sexual orientation, contrasting explanations have been advanced to explain its predictability from facial images, such as self-presentation differences across people with different sexual orientation^8^ (choice to wear glasses, brightness of the image, etc.) vs. biological predisposition story^7^. The controversy, however, does not undermine the ability of facial analysis to strip away privacy, regardless of whether predictions are based on biological features or on other potential signals, such as makeup, hair style, picture angle and lighting, background, etc.

Given the unprecedented proliferation of facial images through user-generated content online^9–12^ and CCTV surveillance^13,14^, these research findings create a tension where companies may want to use the technology to predict information about individuals, while the public may want to have their privacy protected through new laws and policies. This is not a theoretical concern, as facial analysis technology is already being commercialized, mainly for personalization and ad targeting purposes. Amazon facial analysis API can be used to predict from a face gender, age, and emotional expressions, such as surprise, sadness, or happiness^15^. Face-Six provides technology used across US malls to predict customer demographics such as age, gender, and ethnicity^16^, which can then be utilized to show targeted ads on the spot. HireVue provided facial analysis service for job interview decision support, but subsequently stopped following some public backlash^17^. TikTok video platform, which, reportedly, has already reached one billion active users^18^, stated it may collect biometric data, such as ‘faceprints’, from its users’ videos for demographic classification and for content and ad recommendations^19^.

At the same time, individuals enjoy little legal protection against facial analysis. In the US in particular, taking photos or videos without sound in locations where there are no expectation of privacy and no special restrictions by the property owners is almost universally permitted without consent^20^. Scraping of publicly posted facial images is already a common business strategy, which falls into a legally gray area, with some recent court decisions going against the critics of such a practice^12,21^. Existing legislative initiatives primarily target facial recognition—and miss facial analysis. One such recent bill (Commercial Facial Recognition Technology Act of 2019) would make it illegal for a company to create a unique identifier connected to a customer’s facial data without obtaining customer’s explicit consent first^22^. Facial analysis, which, it could be argued, is similar to a keen inspection of an image by an individual, does not necessarily involve creation of an explicit unique identifier for the person in the photo, and could pass under the radar of such a law, remaining unregulated. Similarly, in May 2019, ordinance by the City of San Francisco banned city departments from using facial recognition technology, but not facial analysis^23^.

With the growing use of automated facial analysis, regulators face a decision on whether and how to regulate the technology. Unfortunately, there are problems with the existing literature on the capabilities of facial analysis, hindering policy makers’ ability to conduct a thorough risk-benefit analysis of the technology. Studies in the field tend to assess the predictability from facial images of a single variable or a small set of variables at a time. Different data types, evaluation conditions, and possible confounders across studies complicate use of reported prediction accuracy results to appraise performance of real life facial analysis systems based on machine learning. For instance, images collected in controlled standardized conditions^2^ could yield more consistently styled facial images than selfies sourced online ‘in the wild’ but could also contain fewer signals like makeup, making it hard to generalize results of studies that use such curated images. Differences in whether humans or algorithms are used to extract facial features^24^ and to make predictions^5^ also complicate the comparison and generalizability of results across studies. Another serious issue due to the high-stake focus on one or a few variables per paper is potential selection bias. On the one hand, researchers may investigate only variables they expect to be predictable^25^, so some privacy risks may remain undetected, underestimating the risk of facial analysis technology. On the other hand, variables that are not predictable are likely not to get reported due to the ‘file drawer’ problem^26^, which could lead to exaggerated perceived risk of the technology in the published corpus of works. As a result, we have an upsetting state of knowledge, where a comprehensive ranking of personal data types by their predictability from facial images cannot be reliably constructed based on existing literature, so it is mostly unclear which types of personal information are at a relatively higher or lower risk of exposure through facial images.

In this work, we try to address these limitations by performing a megastudy^27^ that simultaneously investigates the predictability of numerous personal attributes of individuals from their facial images, yielding comparable prediction accuracy scores across 349 diverse variables and their ranking by predictability. We assess the accuracy of predicting personal information from facial image pixels using only deep image features extracted from pixels by neural nets (Figures 2 and 3, Supplementary Table S1). We also investigate how incorporating facial image information incrementally boosts prediction quality compared to a demographics-only benchmark model, considering prediction improvements from basic face metrics like face width-to-height ratio as well as deep image features extracted by neural nets (Figure 4, Table 2). Selection bias concerns are addressed via inclusive approach to selection of predicted personal attributes and by reporting both predictable and unpredictable variables. Further, simultaneous observation of many personal attributes helps us better investigate the mechanism of information signaling by facial images.

## Methods

### Survey data

Our study focuses on prediction of personal data from the facial images of survey respondents. The data for the study was collected via Qualtrics panel and MTurk from November 2018 to February 2019. Each individual was required to respond to a 30-40-minute questionnaire asking varied information about demographics and psychographics and submit three distinct personal facial images. The order of the questions and question options was randomized, where applicable. The study has been approved under Columbia University IRB protocol AAAS1230. All methods were carried out in accordance with relevant guidelines and regulations. Informed consent was obtained from study participants to release their data, including their facial images, in the publication.

#### Question selection

Given that commercial use of facial analysis has focused so far on marketing applications, in planning the questionnaire, we wanted to ask a range of sample questions covering data types that marketers are typically interested in. Unfortunately, a universally accepted typology of data collected and used by marketers does not exist, so we needed to first construct one so that it could guide questionnaire creation. Building on our own experience, several informal interviews with marketing research practitioners and academics, some professional surveys available to us, and a review of frequently used marketing scales^28^, we came up with a rough classification of data types frequently sought by marketers—either actively through surveys, or passively in the course of business operations—and with a large pool of questions representative of these data types. See Table 1 for a summary of the resulting data classification. While we aspired to develop a clear and systematic data typology, we recognize that the proposed classification is inherently not completely sharp. Some types of data could be placed in several categories, for example, gender could be viewed as both a biological (demographic) variable and a psychological (psychographic) variable. Additionally, the classification is not fully exhaustive, especially in the consumption psychographics section. Despite the drawbacks, it proved to be a useful general map of data types for the purposes of this paper. When building out the survey question pool, we tried to optimize for good coverage of the constructed data typology, without regard for our prior expectation about whether specific variables would be predictable from facial images—we hoped that our typology-driven approach would counteract the potential bias / tendency to select variables that are expected to be predictable and would allow for discovery of unexpectedly predictable characteristics.

**Table 1:** Proposed typology of data collected by marketers about consumers.

Category	Sub-category (non-exhaustive)	Examples (could span multiple categories)
Demographics *Traditional socio-biological population characteristics*	Biological characteristics	Gender; race; age; body fitness; employment status; education achieved; household income
	Socio-economic status	
General psychographics *Core psychological and behavioral characteristics*	Character and ethical choices	Big 5 personality: neuroticism; regularly felt emotions: stress; importance of being beautiful vs. being smart; religiosity; active lifestyle; answers to ethical questions, e.g. “What right does your friend have to expect you to lie in court to protect him?”
	Emotional and cognitive state	
	Lifestyle	
	Personality	
	Values and beliefs	
Consumption psychographics *Psychological and behavioral characteristics with respect to specific products/services, brands, ads, categories, proposals, concepts, ideas*	Stated and revealed preferences	Instagram use; preference for iPhone vs. Galaxy; perception of bias: FoxNews; likelihood of recommending Netflix to a friend; preference for Beatles vs. Michael Jackson; price sensitivity: sneakers
	Choice motivations	
	Usage, ownership, and consumption patterns	
	Awareness and recall	
	Reactions, attitudes, opinions, satisfaction	
	Pre-acquisition search	
	Acquisition mode	
	Influence/following	
	Purchase intentions	
	Switching and churn	
	Economic outlook	
	Price sensitivity	
	...	

Note that we exclude some variables as prediction targets in this study when they are objectively and straightforwardly measurable from facial image pixels (even though such variables could be described as ‘personal information’). Examples of such variables include gaze direction, clearly defined facial expressions, eye-wear presence, skin color measured from pixel values, face width, etc. The reason for their exclusion is that such variables could be theoretically resolved to an arbitrarily high precision from pixel values if the face is clearly visible, so the question about the quality of their prediction from facial images seems ill-posed in our study settings.

#### Data preparation

As part of data pre-processing, we filtered out all individuals with incomplete questionnaires, including those without three valid different facial images (in Qualtrics panel data, over a quarter of otherwise completed questionnaires contained invalid images—for example, images of random objects, images that were not distinct, or photos of celebrities; in MTurk data—under 10%). Invalid image submissions could be attributed to reluctance to share personal images—or to automated bot responses, however, it is hard to distinguish these causes and, thus, to know to what degree such filtering biases or unbiases the sample.

We used dlib library^29^ to automatically extract aligned 224$\times$224-pixel face squares, where possible. Continuous and multilevel categorical response variables were binarized—via one-vs-all binarization or by splitting scales—to allow for consistent prediction quality comparisons. For example, multilevel ‘Gender’ variable was split into separate ‘Male’, ‘Female’, and ‘Other’ binary indicators (where 1 indicates the focal gender level, while 0 indicates reference gender levels). As a result, 349 binary response variables were extracted from each completed questionnaire to be used as prediction targets.

In the end, we have total n=2646 paired image-response observations (969 individuals, $\sim$76% female): 2312 observations from Qualtrics (853 individuals, $\sim$82% female) and 334 observations from MTurk (116 individuals, $\sim$37% female). Sample facial images are shown in Supplementary Figure S1, variable-level summary statistics are provided in Supplementary Table S1.

Principal component analysis of 349 response variables suggests a complex correlation structure, which cannot be summarized well by just a few factors. For illustration, the first 5 principal components explain only 17% of variance, 50 principal components explain 52% of variance, and 195 principal components are required to explain 90% of variance.

### Analysis methodology

**Figure 1:**
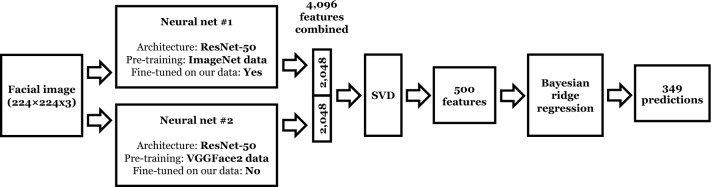
Prediction pipeline: a facial image is processed by two distinct ResNet-50 neural nets, followed by SVD, followed by the Bayesian ridge regressions predicting 349 binary variables.

The first question we address is how well each binary survey response variable can be predicted from facial image data alone using a powerful binary classifier. The prediction pipeline (sequence of data processing algorithms, where the output of one algorithm is the input of the next one) involves two deep convolutional neural nets each extracting 2048 features from a $224\times 224$ image with 3 color channels, followed by singular value decomposition (SVD) that is used to reduce the combined 4096 features output by the two neural nets to 500 features, and then a separate Bayesian ridge regression (scikit-learn Python library implementation)^30^ relating each binary response variable to the 500 extracted features. Use of SVD is meant to reduce collinearity between deep image features extracted by the neural nets^7^. Bayesian ridge linear regression automatically selects the optimal L2 regularization strength in the estimated linear probability model for each binary prediction. Both neural nets use an advanced ResNet-50 deep learning architecture^31^, one pre-trained on 1000-class ImageNet dataset^32^, and its last several layers fine-tuned on our data to simultaneously predict target variables (similar to the multi-label net^33^); another pre-trained for facial recognition on VGGFace2 data set^34^ and not fine-tuned on our data. We have found that the fine-tuned ImageNet model boosts predictive power of the images beyond demographic variables, whereas addition of VGGFace2 model enhances the accuracy of demographic predictions. This neural net setup is tailored to our relatively small data sample, which is insufficient to train a full image-processing neural net from scratch, so instead we take the approach known as transfer learning^35^, where we start with a model pre-trained on a large data set. Figure 1 illustrates the full prediction pipeline from an image to 349 binary response variable predictions.

To measure holdout quality of a binary classifier, we use the standard AUC metric (area under the ROC curve), calculated across observations (separate images). AUC captures the relative frequency with which a randomly drawn positive observation is assigned by the classifier a higher probability than a randomly drawn negative observation. An advantage of AUC is that it is robust to imbalanced data, as it does not utilize a specific prediction threshold. AUC is 1.0 for a perfect classifier and 0.5 for a random one. To ensure reliability of AUC estimates, we conduct repeated 5-fold cross-validation^36,37^: 20 times we randomly split the whole data into five disjoint sets, iteratively using one of the five sets for testing, after training the full prediction pipeline—neural nets, SVD, and Bayesian ridge regressions—on the other four. This procedure yields k=100 ($20\times 5$) AUC measures per classifier. (If a variable is sparse and its realization is unobserved in a given test fold, AUC cannot be calculated, so k could be lower than 100; this is rare, but occurs, e.g., for ‘Gender: Other’ variable.) Cross-validation split is at the individual level—any individual with all their pictures is in one of the five folds, but not in any other.

Sampling distribution for AUC statistic is approximately Normal^38^. We estimate the distribution’s mean and standard deviation (i.e., AUC standard error) for each tested variable from AUC values observed in cross-validation runs. Intuitively, the more likely AUC values for a variable are to fall above 0.5, across different data and algorithm realizations, the more confident we are the variable is predictable better than random. When the lower bound of the AUC two standard error (2SE) confidence interval is above 0.5, we say that a variable is predicted significantly better than random based on 2SE criterion. (The word ‘significantly’ should be interpreted with some caution considering the biasedness of cross-validation variance estimators^39^.)

To keep in check the number of false positives under multiple testing across variables, we further use a false discovery rate method that ensures the expected false discovery rate—expected proportion of rejected cases (null hypothesis $\hbox {H}_0$: AUC $\le$ 0.5) that are wrongly rejected—is under a specified level q=0.05. For each predicted variable, we use the cross-validated AUC mean and standard error to calculate a p-value—an area to the left of AUC=0.5 under Normal CDF. We then apply Benjamini-Hochberg (BH(q)) step-up procedure^40^, obtaining a conservative adjusted p-value cutoff threshold for rejection of $\hbox {H}_0$ for the set of evaluated variables. Let *tp* (true positive) denote the number of correct rejections of $\hbox {H}_0$ and let *fp* (false positive) denote the number of incorrect rejections of $\hbox {H}_0$, based on some p-value threshold. Use of the BH(q) adjusted p-value threshold to reject $\hbox {H}_0$ ensures $E[fp/(tp+fp)]\le q$. The procedure is valid even in case of dependence between evaluated variables, under empirical Bayes view of BH(q)^41^.

See Supplementary Information for more details on data collection and pre-processing, model training, and inference.

**Figure 2:**
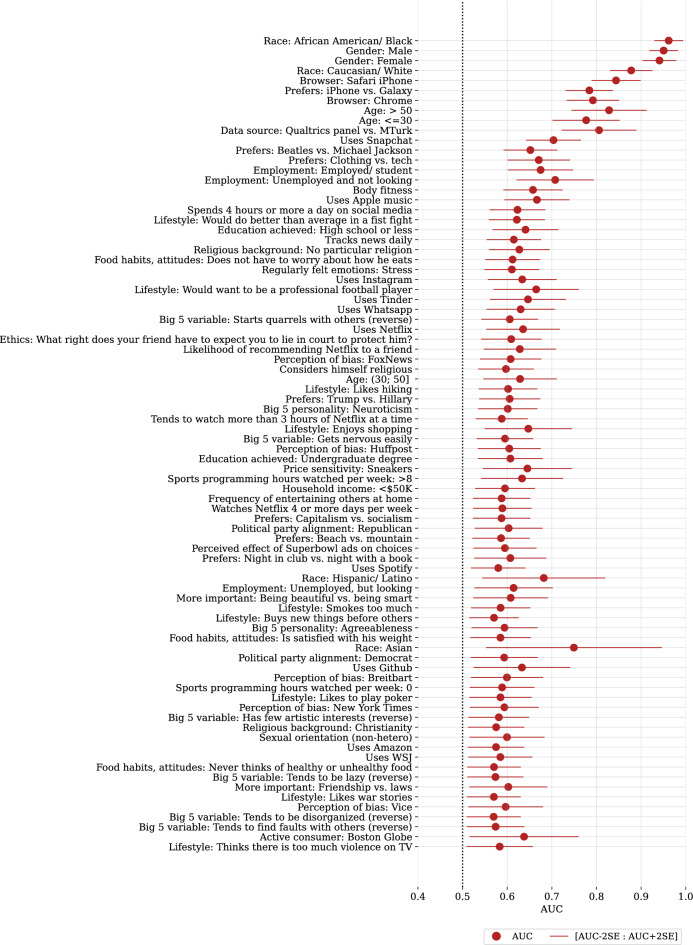
Cross-validation results for 82/349 ($\sim$23%) personal attributes, where binary predictions from facial images beat random guess (mean holdout AUC values are above 0.5 random threshold) based on BH(q=0.05) statistical significance criterion. Predictions are from a Bayesian ridge regression, based only on deep image features extracted from facial image pixels by neural nets. Variables are sorted in increasing order of p-values. AUC means and 2SE intervals are estimated from k=100 holdout AUC measures.

**Figure 3:**
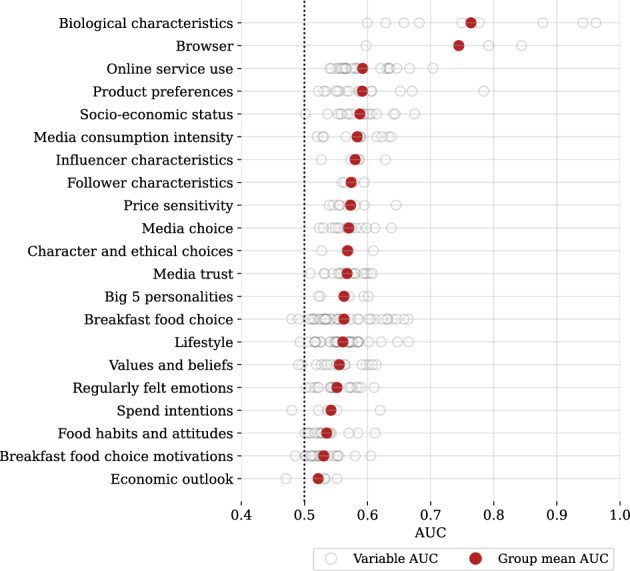
Predictability of variable categories from facial images, without regard for significance. Predictions are from a Bayesian ridge regression, based only on deep image features extracted from facial image pixels by neural nets. Group mean AUC is calculated as an average of cross-validated AUC estimates for the variables in the group. This classification is derived from Table 1, but contains somewhat narrower categories corresponding to the specific questions included in the questionnaire.

## Results

### What information is predictable from facial images?

Figure 2 shows the results for $\sim$23% (82/349) variables that are predicted significantly better than random based on BH(q=0.05) procedure with a p-cutoff=0.012 (and thus also based on the less conservative 2SE confidence interval with implied p-cutoff=0.023). BH(q=0.05) procedure ensures the expectation of $\le 5$% of false positives among all rejections (82 here, which implies $\sim$4 expected false positives). All variables are ordered in the increasing order of p-values (decreasing significance). Variables predictable from image pixels based on BH(q=0.05) criterion are varied and include, among others, age, gender, and race; iPhone vs. Galaxy preference; use of Chrome and iPhone Safari browsers (captured in survey metadata); stated Netflix use; preference for Trump vs. Hillary; expression of Big 5 neurotic personality; and even an ethical judgment in response to a question “What right does your friend have to expect you to lie in court to protect him?” from the Car and the Pedestrian experiment^42^. Many predictable variables, such as browser type, Apple Music use, religious background, and employment status, initially surprised us, and we would not have picked them as our focal variable in a single-variable study of predictability from facial images, highlighting the value of testing multiple hypotheses simultaneously for knowledge discovery. Figure 3 provides a systematic overview of predictability for variables grouped by data type. Biological and socio-economic demographics, product and service use and preferences tend to be more predictable from facial images, while such data categories as regularly felt emotions, lifestyle, and economic outlook are harder to predict. Supplementary Table S1 gives results for all variables, predictable or not.

**Figure 4:**
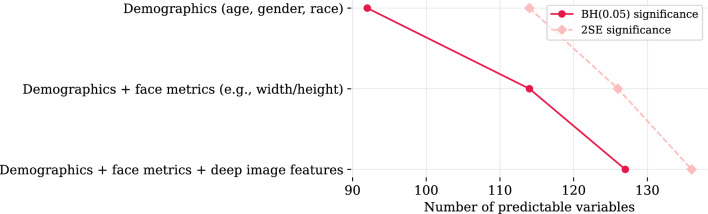
Number of variables (out of total 349) predicted significantly better than random, depending on the set of input variables in a Bayesian ridge regression. Perfect knowledge of demographics is a powerful predictor, with 92 BH(q=0.05) significantly predicted variables. Addition of facial image information—manually calculated basic face metrics plus features extracted via deep learning from the facial image—boosts the number of predicted variables by $\sim$38% to 127. Without deep image features, basic face metrics deliver a smaller boost of $\sim$24% (114 predicted variables).

#### Controlling for demographics

It is well known that human facial images strongly signal age, gender, and race^1^. Our analysis confirms that these demographic variables are among the strongest signals in facial images. Demographics is also known to be a strong predictor of varied individual behavior^43^, so demographics inferred from facial images could be driving predictions of all other personal attributes in Fig. 2. To understand whether facial images reveal extra personal information, beyond what demographics can predict, we estimate three progressively more complicated models based on (a) basic demographics stated in the survey (age, gender, race); (b) eleven basic face metrics manually computed from facial images, based on dlib facial landmark coordinates (including RGB color channels averaged across face oval and facial width-to-height ratio); (c) deep image features extracted by the deep learning model from facial images. Specifically, we track the number of predictable variables, as we sequentially expand the information set input into the Bayesian ridge regression—from demographics to basic face metrics, to facial features extracted by the neural nets from facial images. See Supplementary Information for details on demographic controls and the full list of basic face metrics. Figure 4 summarizes our findings. We find that stated demographics alone forms a powerful predictor across a variety of data types. However, basic metrics calculated from facial features as well as deep image features extracted by neural nets all expand the set of predictable variables beyond what knowledge of only demographics allows for. In particular, while the reference demographics-only model BH(q=0.05) significantly predicts 92 variables, adding all information based on facial images to the model boosts the number of predicted variables by substantial $\sim$38% to 127. Thus, with known demographics, information in facial images adds extra 35 variables to the predictable set. Average AUC across 349 variables also increases with additional model input—from 0.577, to 0.579, and to 0.586 respectively. Compared to the number of significantly predicted variables, average AUC across 349 variables shows a more muted increase with expanded model input, as it is weighed down by most variables not being significantly predictable from any of the considered input sets (AUC$\approx$0.5).

**Table 2:** Increase in mean holdout AUC as we incrementally add more inputs to a reference demographics-only Bayesian ridge regression.

No.	Variable	% Increase, AUC	Ref. AUC	New AUC
I. Basic face metrics are added—to demographics				
1.	Body fitness	8.0%	0.61	0.66
2.	Food habits, attitudes: Is satisfied with his weight	6.7%	0.60	0.64
3.	Prefers: iPhone vs. Galaxy	4.7%	0.59	0.62
4.	Is a frequent alcohol consumer	4.6%	0.56^†^	0.58
5.	Browser: Safari iPhone	4.5%	0.66	0.69
6.	Considers himself religious	4.1%	0.58^†^	0.60
7.	Likelihood of following a movie recommendation from a friend	3.8%	0.59^†^	0.61
8.	Perception of bias: BBC News	3.7%	0.60^†^	0.63
9.	Religious background: No particular religion	3.4%	0.62	0.64
10.	Food habits, attitudes: Does not have to worry about how he eats	3.0%	0.59	0.61
11.	Prefers: Madonna vs. Lady Gaga	2.9%	0.56^†^	0.58
12.	Active consumer: BBC News	2.9%	0.59^†^	0.61
13.	Prefers: Chocolate ice cream vs. strawberry ice cream	2.8%	0.55^†^	0.57
14.	Uses Telegram	2.8%	0.64	0.66
15.	Sexual orientation (non-hetero)	2.6%	0.63	0.65
16.	Spends 4 hours or more a day on social media	2.4%	0.63	0.64
17.	Big 5 variable: Starts quarrels with others (reverse)	2.2%	0.62	0.63
18.	Food habits, attitudes: Never thinks of healthy or unhealthy food	2.1%	0.57^†^	0.58
19.	Political party alignment: Independent	1.8%	0.58^†^	0.59
20.	Religious background: Christianity	1.8%	0.60	0.61
21.	Perception of bias: FoxNews	1.8%	0.59	0.60
22.	Prefers: Clothing vs. tech	1.6%	0.68	0.70
23.	Big 5 personality: Agreeableness	1.6%	0.65	0.66
24.	Lifestyle: Likes hiking	1.6%	0.60	0.61
25.	Browser: Chrome	1.5%	0.61	0.62
II. Deep image features are further added—to demographics and basic face metrics				
1.	Browser: Chrome	28.2%	0.62	0.79
2.	Prefers: iPhone vs. Galaxy	25.7%	0.62	0.78
3.	Browser: Safari iPhone	22.7%	0.69	0.85
4.	Employment: Unemployed, but looking	15.6%	0.54^†^	0.62
5.	Lifestyle: Smokes too much	13.5%	0.52^†^	0.59
6.	Uses Apple music	10.9%	0.60	0.66
7.	Actively recommends movies to watch to friends	6.7%	0.54^†^	0.58
8.	Data source: Qualtrics panel vs. MTurk	6.5%	0.77	0.83
9.	Uses WSJ	6.0%	0.55^†^	0.58
10.	Prefers: Night in club vs. night with a book	5.7%	0.57^†^	0.60
11.	Education achieved: High school or less	5.5%	0.63	0.66
12.	Household income:<$50K	5.5%	0.57	0.60
13.	Education achieved: Graduate degree	4.9%	0.58^†^	0.61
14.	Sports programming hours watched per week: >8	4.4%	0.63	0.65
15.	Prefers: Original coke vs. diet	4.3%	0.57^†^	0.60
16.	Employment: Employed/ student	3.9%	0.68	0.71
17.	Uses Netflix	3.7%	0.63	0.65
18.	More important: Friendship vs. laws	3.5%	0.57^†^	0.59
19.	Likelihood of recommending Netflix to a friend	3.4%	0.63	0.65
20.	Uses Amazon	3.2%	0.56^†^	0.58
21.	Body fitness	2.6%	0.66	0.68
22.	Food habits, attitudes: Does not have to worry about how he eats	2.5%	0.61	0.62
23.	More important: Being beautiful vs. being smart	2.5%	0.59^†^	0.60
24.	Household income: [50*K*, 100K)	2.4%	0.55^†^	0.57
25.	Big 5 variable: Tends to be disorganized (reverse)	2.3%	0.56^†^	0.58

Showing top 25 variables by AUC increase. The dagger symbol ($\dagger$) marks AUC values that are not BH(q=0.05) significant. This table shows improvements where new AUC is significant by BH(q=0.05) criterion and increase in mean AUC is significant at 0.05 level based on one-sided two-sample t-test with unequal variance.

AUC values are rounded to two decimal places for presentation purposes.

Table 2 shows variables that benefit the most in terms of prediction accuracy from addition of facial image features to demographic information. Addition of basic face metrics boosts our ability to predict reported body fitness level, individuals’ satisfaction with their weight, tendency to think about healthy vs. unhealthy food, alcohol consumption, religiosity, sexual orientation, consuming media content in large quantities, and quarrelsome personality, among other variables. Further addition of deep image features boosts our ability to predict the browser used by the consumer when completing the survey (iPhone Safari vs. Chrome), iPhone vs. Galaxy preferences, employment status, smoking habits, household income and education levels, Apple, WSJ, and Amazon use, etc. Table 2 also shows the variables that are not BH(q=0.05) significantly predictable from demographics alone and only become predictable with addition of facial image information—these include alcohol consumption frequency, smoking habits, religiosity, ‘unemployed, but looking’ job status, as well as some media content consumption patterns and food habits.

#### Observed patterns and their possible explanations

We have so far presented evidence that facial images signal personal information beyond what demographics alone can reveal. Some high-level patterns emerge. First, our model can incrementally predict the fitness of the individuals and their (un)healthy habits, such as smoking and alcohol consumption, from the facial images. Prior research has established that body-mass index (BMI)^44^; smoking and alcohol use^24^ all correlate with facial appearance, indicating external validity of this result. Second, facial images signal social class characteristics such as individual’s unemployment status and education level. Prior research has suggested the existence of correlation between facial cues and social class, including employability perceptions^4^—our results support these findings. Third, facial images seem to signal the internal mental states of individuals, as reflected, for example, in religiosity and high volume media content consumption. This is also a conceivable result. Facial hair could be indicative of religious observance, for example, in Judaism and Islam. Further, prior research has established some link between BMI and screen media use^45^, BMI and depression^46^, BMI and religiosity^47^, alcohol consumption and depression^48^, and depression and religiosity^49^, which could represent some of the plausible pathways for facial images to reveal the mental state information.

Interestingly, facial images also contain a very strong signal about the browser used by the respondent—for instance, use of facial images boosts the prediction accuracy of iPhone Safari browser variable to AUC 0.85 vs. demographics-only model AUC of 0.66—the boost being driven primarily by the deep image features (Table 2). This signal could result from camera artifacts imposed on the images, post-processing by smartphone-specific software, or human behavior differences in smartphone owners and audiences. It has also been reported in the literature^50^ that a given smartphone can be *uniquely* identified based on a single image—due to a unique pattern of photo-response in every camera’s image sensor. Brand-specific photo-response patterns could explain our ability to predict browsers and smartphone preferences from facial images, but we are not fully certain if this is what the neural nets are picking up. However, if future research can confirm that images can be uniquely attributed to a specific smartphone via the camera photo-response fingerprint, this would have wide implications for our ability to uniquely attribute all the images back to their authors and track consumer activity online, with potentially grave privacy implications for image takers who want to preserve the anonymity of their work.

A likely explanation for this variety of significant signals is simply that the personal characteristics tend to be correlated with each other. For example, characteristics such as body fitness, skin health, or facial hair, as well as user’s browser/device, which are more plausibly predictable from facial images, can be correlated with numerous more complex personal characteristics such as social class, religiosity, consumer behavior, and media consumption. It is thus plausible that facial images could signal these higher order personal characteristics in such an indirect manner, even if one may view such findings as surprising at first. To illustrate the point, Supplementary Table S2 shows the Pearson correlation of the body fitness and iPhone Safari browser variables with 30 variables that are most significantly predictable based on deep image features alone. We find the body fitness is strongly correlated with employment status, which offers a plausible pathway for inferring the employment status based on a facial image—such prediction would not be perfect, but the statistical signal is there. Body fitness is also correlated with Tinder use, not having to worry about what one eats, and overall lower levels of stress, which could thus also be plausibly inferred via facial images. Prediction of iPhone Safari browser is also informative about other variables—it signals increased likelihood of Snapchat and Apple Music use, a more likely preference for iPhone over Galaxy phone, and informs about user’s preference for clothing vs. tech. Thus, prediction of a few facts about a person via facial images can lead to a wealth of inferences about them.

#### Limitations

The following caveats apply. Imperfections in image pre-processing could add noise. Binarization of continuous variables may underestimate their predictability^51^. People control what pictures they submit, so prediction may be driven not just by true appearance, but also by how people choose to look online, the edits they make to the photos, or camera/device artifacts left in the image (this is generally the case for personal user-generated content uploaded online, so is not strictly a limitation as far as generalizability of results to such content is concerned). This also means CCTV images may contain less information than selfies, so our study better characterizes the latter. People might lie or err when submitting survey responses. Results are primarily correlational in nature. Results are true for the present sample of panel participants (in particular, individuals who were comfortable with sharing their images and personal responses with us) and do not necessarily generalize to the population. The sample size is relatively small. Prediction is based on a single image—forecasts from multiple images for the same person are not aggregated. Models we have not considered could offer improved predictive power. Overall, the study likely provides a lower bound on prediction quality of facial analysis—companies could do better across data types with larger samples, use of revealed preference data, by aggregating over different images of the same person, by dropping binarization of continuous variables, and by further fine-tuning the predictive models. Note that we focus on predictions from image pixels and exclude from the scope of our study image metadata (text information associated with the image, which could contain geolocation, camera device description, and other personal information)—the metadata can present significant privacy risks, but, compared to data captured in image pixels, it can be relatively easily erased by the user, without altering the image itself^52^.

### What parts of facial images signal the information?

One interesting and important question is what specific parts of a facial image function as the information source for predictions. Understanding the origin of these signals could provide face validity evidence (no pun intended) for our findings. We can hypothesize that some signals should be straightforward to explain. Width of the face could indicate the level of body fitness. Facial skin color could suggest race. Such signals could be inferred from specific local areas of the facial image. Other signals, such as the browser used by the respondent, to the extent they are driven by pixel-level artifacts, could be imperceptible to a passing visual examination—they could be scattered all over the image or concentrated in specific areas. We devise three approaches to empirically examine which facial image areas originate different signals. First, we explore the correlations between selected face metrics and predicted variables. Second, we track which areas of an image, when blocked, lead to the greatest decrease in AUC in predictions based on deep image features. Third, we estimate a novel model based on L1 regularization that identifies average linearly decomposable visual imprints of different features on the facial image.

**Table 3:** Pearson correlations between selected face metrics and predicted variables.

No.	Variable	Face color			Face shape			Deep PC
		R	G	B	W	H	W/H	Smoking
1.	Race: Caucasian/ White	**0.18**	**0.17**	**0.22**	0.04	0.02	0.04	0.01
2.	Race: African American/ Black	**− 0.22**	**− 0.22**	**− 0.24**	− 0.06	− 0.06	0	− 0.02
3.	Gender: Male	**− 0.19**	**− 0.18**	**− 0.16**	− 0.05	**− 0.09**	**0.14**	**− 0.1**
4.	Gender: Female	**0.19**	**0.18**	**0.16**	0.04	**0.08**	**− 0.13**	**0.1**
5.	Age: $<=$30	**0.13**	**0.15**	**0.12**	0.07	**0.1**	**− 0.11**	**− 0.08**
6.	Age: >50	− 0.06	**− 0.09**	**− 0.07**	− 0.06	**− 0.11**	**0.16**	**0.18**
7.	Uses Snapchat	**0.11**	**0.14**	**0.12**	0.01	0.05	**− 0.09**	0.01
8.	Browser: Safari iPhone	**0.12**	**0.1**	0.05	**0.14**	**0.13**	− 0.01	**− 0.1**
9.	Prefers: iPhone vs. Galaxy	**0.09**	0.04	− 0.01	**0.1**	**0.09**	0.01	**− 0.08**
10.	Employment: Unemployed and not looking	0	− 0.01	0	− 0.03	**− 0.08**	**0.13**	**0.09**
11.	Body fitness	− 0.03	− 0.01	− 0.03	**− 0.12**	− 0.06	**− 0.15**	− 0.02
12.	Food habits, attitudes: Is satisfied with his weight	**− 0.09**	**− 0.08**	**− 0.08**	**− 0.16**	**− 0.12**	− 0.06	0
13.	Considers himself religious	− 0.01	0	− 0.02	− 0.06	**− 0.1**	**0.13**	**0.12**
14.	Sexual orientation (non-hetero)	0.05	0.05	0.05	**0.08**	0.06	0.03	− 0.03
15.	Big 5 variable: Starts quarrels with others (reverse)	− 0.04	− 0.02	− 0.02	**− 0.07**	**− 0.09**	0.06	0
16.	Big 5 variable: Gets nervous easily	**0.07**	**0.09**	**0.08**	0.01	0.01	0.02	0.02
17.	Lifestyle: Smokes too much	− 0.04	− 0.02	0	− 0.04	− 0.01	− 0.07	**0.12**
18.	Is a frequent alcohol consumer	− 0.05	− 0.03	− 0.03	− 0.04	0	− 0.07	− 0.02

Face metrics include RGB color channels averaged across face oval; facial width, height, and width-to-height ratio (fWHR); and a deep image feature (principal component) with the highest correlation with the smoking frequency variable. Correlations are highlighted when significant at two-tailed significance level 0.01 using individual-clustered standard errors.

#### Correlations between face metrics and predicted variables

Table 3 presents correlations between selected representative face metrics and predicted variables. We can observe an abundance of weak correlation patterns. Facial pixel luminosity (brightness) across color channels signals race, gender, age, weight satisfaction, and proclivity to get nervous easily. Red pixel luminosity in particular is weakly but significantly correlated with iPhone browser use and preference for iPhone over Galaxy by the respondent. Facial shape weakly signals gender, body fitness, and quarrelsome personality. As an experiment, we also extracted a deep image feature (principal component) with the highest correlation with the smoking frequency variable. This feature additionally signals religiosity, age, employment status, and preference for Galaxy over iPhone—it thus seems to be capturing a person’s facial ageing. Overall, these correlations all seem plausible and showcase how even simplest facial color and shape metrics contain personal information hints. It is also worth noting that the multitude of small correlations across different sources can compound to form an even stronger signal.

#### Prediction-critical facial image areas

**Figure 5:**
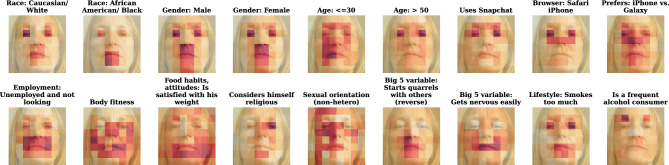
Importance of image segments for the quality of prediction of selected variables from deep image features alone. Heat map indicates relative magnitude of AUC decrease for a variable when a square area of an image is masked (more intense color means greater decrease), avg. across images and CV folds. Background is a selected representative image.

To get more insight into the mechanism by which facial images reveal information, we examine facial image areas that are most critical for quality of the predictions based on deep image features. We sequentially block segments of an image on an 8$\times$8 grid to measure AUC decrease compared to when the full image is visible, averaged across images and cross-validation steps. Figure 5 shows corresponding heat maps for selected variables. See Supplementary Figure S2 for more variables. Localized eye and mouth information seems to be of importance in predicting many variables, including gender and browser/device use. As another example, for predicting whether a person gets nervous easily, the mouth area tends to be particularly important. For variables such as body fitness and weight satisfaction, simultaneous access to information across the face oval seems to be relatively more important to achieve high-quality predictions. These results indicate the importance of specific local facial features for prediction. In principle, the downside of this approach is its narrow focus on image areas that contain unique (non-redundant) information. If information is redundantly stored all over the image, blocking an image segment should not prevent the neural nets from accessing that same information elsewhere—and the corresponding area would not be highlighted as important, even though it can serve as an information source. For this reason, this approach is unlikely to detect redundantly encoded image properties that can inform prediction.

#### L1-regularized facial image decomposition into feature-specific imprints

**Figure 6:**
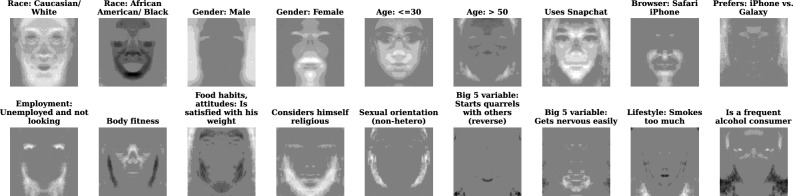
Selected coefficient matrices $P_j$ from a regression of facial images *X* on binary features *Y* under L1 regularization. Lighter color indicates more positive coefficient values, darker color—more negative coefficient values, and gray color indicates values close to zero. Coefficients have been normalized for visualization: $P_j/\max (|P_j|)$. The coefficient matrices represent visually prominent imprinting of different features on consumer facial images, as identified by the linear model.

To dig deeper into the question of what image areas signal personal information, we propose a novel lasso-based model to learn facial prototypes associated with each binary feature in the data. Consider a model of a $224\times 224$ facial image *X* (in gray scale), where it is approximated as a sum of prototypical $224\times 224$ images $P_j$ corresponding to a set of 349 known binary features $y_j$ ($P_0$ corresponds to an ‘intercept’ image). *X* and $y_j$ are given. $P_j$ are unknown and have to be estimated to get $X \approx P_0 + \sum _{j=1}^{349} P_j\cdot y_j\text {.}$ In other words, we are decomposing *X* into 349 $P_j$ matrices (imprints) that reflect the average contribution of each $y_j$ to the construction of *X*, plus the intercept. We can estimate parameters $P_j$ by solving the following optimization problem across *n* observations in our data:

$$\begin{aligned} \min _{P_j \forall j\in 0:349}\frac{1}{n}\sum _{i=1}^n||X_i - P_0 - \sum _{j=1}^{349} P_j\cdot y_{ij}||^2_F+\lambda \sum _{j=0}^{349} \textbf{1}^T|P_j|\textbf{1}{} \textit{,} \end{aligned}$$

where $\lambda$ indicates regularization strength, $\textbf{1}$ is a vector of ones, $||\cdot ||^2_F$ denotes a Frobenius norm, and $|\cdot |$ is element-wise absolute value. The use of L1 regularization encourages the redundant parameters to be set to exactly zero. If a given feature $y_j$ is uncorrelated with the facial image appearance, then the whole corresponding matrix $P_j$ should be set zero. The model involves millions of parameters—we use the highly scalable OWL-QN algorithm for L1-regularized optimization^53^ to perform the inference (we use the following Python library: https://bitbucket.org/rtaylor/pylbfgs/src/master/). To aid the estimation, we use reparametrization to enforce a symmetry restriction, where the left side of a coefficient matrix is equal to its horizontally flipped right side $P_j[:,0:112,:] = {\textbf {flip}}(P_j[:,112:224,:])$, which cuts the number of estimated parameters by half. We set $\lambda =0.01$ for moderate regularization strength and continue training until ten sequential iterations fail to yield a decrease in the loss, which happened after 644 OWL-QN iterations. We found $\lambda =0.01$ strikes the detail vs. simplicity balance in the $P_j$ visualizations (Fig. 6).

The estimated matrices $P_j$ indicate the visually prominent linearly decomposable ‘fixed’ effects of different features on the facial images. This analysis capitalizes on our tracking multiple personal attributes simultaneously, so each effect is estimated while ‘controlling for everything else’. Importantly, non-linear dependencies between images and variables (for example, due to higher-order interactions between pixels in different parts of the image, or because a prominent visual effect occurs only when features are interacted) would not be captured by this procedure. Nevertheless, $P_j$ matrices can help us understand the relative role of the visually prominent facial features in signaling personal information. We visualize the estimated coefficient matrices for selected variables in Fig. 6. See Supplementary Figure S3 for more variables. Positive numbers in $P_j$ signify an increase in corresponding pixel luminosity (whiter color), negative numbers indicate a decrease in luminosity (darker color), and zeros imply no effect. Some results, arguably, make intuitive sense—for example, lighter image is associated with a white race, a darker image is associated with an African American/black race. The prototypical images corresponding to body fitness and weight satisfaction have cheek areas blacked out. The prototypical image corresponding to self-perception of religiosity has a beard/chin-and-jaw area highlighted. Other images can be tougher to interpret. For instance, the image associated with getting nervous easily has a mouth area highlighted, mimicking the result in Fig. 5, but it is unclear what drives this result.

Notice that the absolute value of a prototypical image $|P_j|$ indicates areas in the image that experience change under the $y_j$ feature variation. We propose a novel quantity, which we call a *visual prominence score* $v_j=\textbf{1}^T|P_j|\textbf{1}$. High $v_j$ indicates high magnitude/prominence of the visual contribution of a feature to a facial image. We hypothesize that features with high visual prominence should also be more predictable from the facial image. Indeed, we find a high Pearson correlation between vector *v* of visual prominence scores and the hold-out AUC of the 349 features: ${\textbf {corr}}(v,\text {AUC})=0.56$. This suggests that visually prominent features, as determined by this model, are more easily predictable. However, the visual prominence of features in a facial image, thus measured, explains only part of the the feature predictability from facial images ($R^2=0.31$)—the rest of it could be attributed, for example, to color information lost through use of the gray scale or to higher-order interactions between pixels in different parts of the image, not captured by the image decomposition procedure.

## Discussion

Our study is conducted on a relatively small sample (969 individuals, 2,646 distinct facial images) but, to the best of our knowledge, is the largest as of now in terms of the number of personal attributes (349) assessed simultaneously under equivalent conditions for predictability from facial images. Overall, 23% of 349 variables assessed were predictable better than random. Figures 2 and 3 and Supplementary Table S1 provide a first-of-its-kind comprehensive ranking of personal attributes by their predictability from facial images illustrating what personal information is at a relatively higher or lower risk of exposure.

The strongest startling effect we detect, enabled by our generous inclusion of various variables into the assessment, is the high prediction quality from facial images alone of the iPhone Safari browser used by the respondent at AUC=0.84 (variable captured in survey metadata) and the respondent’s preference for iPhone vs. Galaxy phone at AUC=0.78. We hypothesize these indicate our ability to predict the smartphone that took the image from image pixels—possibly, due to sensor or algorithm level artifacts imposed by the smartphone camera. The ability to predict the originating device from image pixels deserves further research due to potentially grave privacy implications if images turn out to be reliably attributable back to devices that took them, leading to deanonymization of image takers who would prefer to keep their identity hidden.

Outside top 20 predictable variables (demographics, smartphone variables, body fitness, etc.), the majority of other significantly predictable personal attributes achieve relatively low prediction accuracy levels around AUC$\approx$0.6. We argue these are representative results on smaller sample sizes, like ours, and on images collected outside of controlled/special conditions (e.g., controlled lab settings that may affect levels of signaling by individuals). In other words, we view our results as a lower bound on what is achievable using facial analysis. We hypothesize there is a substantial accuracy upside that could be achieved for our results through a larger sample size. For instance, while we report AUC=0.6 for sexual orientation prediction, higher AUC of 0.71 (women) and 0.81 (men) has been reported^7^ on larger training data of around 75 thousand individuals and three hundred thousand images. For political orientation (‘Political party alignment: Republican’), while we report AUC=0.6, a study on over a million respondents for a similar variable ‘liberal vs. conservative’ has reported AUC in the 0.7-0.73 range, depending on the evaluation set^5^. (Yet even such large sample sizes do not allow for perfect AUC of $\sim$1.0 on such attributes).

Our simultaneous observation of multiple personal attributes per person facilitates an investigation of the mechanism behind how facial images signal information. Correlation analysis suggests that the most likely explanation for the variety of signals in facial images that we observe is that personal attributes predict each other—for instance, body fitness (which is plausibly predictable from face bulkiness/width in a facial image) turns out to be correlated with employment status in our data and thus offers a plausible mechanism for the surprising employment status predictability from a facial image. Our L1-regularized image decomposition technique reveals specific facial image areas that signal personal information: e.g., cheek areas indicate body fitness and weight satisfaction; beard/chin-and-jaw areas indicate self-perception of religiosity. Visual prominence of the image areas identified by L1 decomposition is strongly correlated with observed AUC scores across 349 variables, supporting the validity of our interpretation of these visual areas as drivers behind the predictions. Overall, our analyses point to smartphone camera artifacts, BMI, skin properties, and facial hair as top candidate non-demographic signals in facial images.

As to implications for policy makers, while there are a few variables that facial images provide a stronger signal for (smartphone type or the more visually obvious demographics or body fitness), we find 77% of 349 considered variables cannot be predicted from facial images better than random, and the rest of the predictable variables achieve only moderate prediction quality at AUC$\approx$0.6. Even if large sample sizes could boost the accuracy to higher levels such as AUC=0.8, observed in larger sample studies^5,7^ for select variables, these evidence levels do not stand as an undeniable proof that a person possesses or does not possess a particular personal attribute. Therefore, if the type of risk we worry about is the revelation of some personal trait via facial image pixels in a way that is definitive, yet non-obvious to human eye, such risk looks limited across most variables.

At the same time, we do find that pixel values of a single facial image provide a lot of weak versatile statistical signals on individual’s behaviors, preferences, character, personality, and beliefs, across a wider range of variables than previously reported. Such weak information signals can be used by rational agents to inform their decisions—more innocently in personalization and ad targeting (ad targeting industry, in particular, is known to utilize weak statistical signals^54^) and far less innocently in hiring or making decisions in court settings. Prejudice caused by decision makers’ inappropriate exposure to such statistical information is thus the main policy concern, in our view. To address this concern, we advocate for the use of blinded procedures when any risk of personal information exposure is unacceptable. The right to be forgotten and similar laws can also help by enabling individuals to force the removal of their facial images and corresponding private information where they so desire.

We hope that our results will stimulate further research in the area of facial analysis and encourage wider use of megastudies that assess multiple variables of interest simultaneously as a tool for knowledge discovery. In our view, potentially fruitful directions for future research include: (a) studying ways to improve facial analysis accuracy overall and, to minimize possible biases, across specific demographics groups^55^; (b) investigating the possibility of attribution of images to the originating smartphone camera device and the privacy implications thereof; and (c) investigating the quality of personal information predictions from more extensive visual information about a person, such as full-body video footage.

### Supplementary Information

Supplementary Information.

## Data Availability

The data and code used to compute the results as well as the questionnaire used in data collection are available at https://github.com/computationalmarketing/facial-analysis-megastudy.
