# Supplementary material for: A megastudy on the predictability of personal information from facial images: Disentangling demographic and non-demographic signals
Source: Sci Rep. 2023 Nov 29;13:21073. doi: 10.1038/s41598-023-42054-9 (PMC10687237; doi:10.1038/s41598-023-42054-9)
Supplement: Supplementary file 1 — Supplementary Information. [file 41598_2023_42054_MOESM1_ESM.pdf]

Supplementary Information

for A Megastudy on the Predictability of Personal Information From Facial Images

Yegor Tkachenko (yegor.tkachenko@columbia.edu), Kamel Jedidi (kj7@gsb.columbia.edu)

Supplementary figures and tables

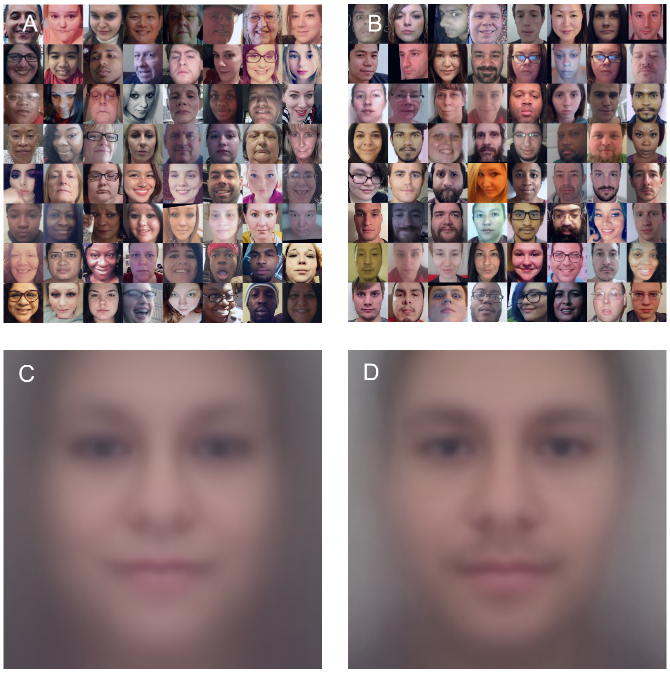

Figure S1. Images of respondents. (A, B) Sample facial images of respondents from Qualtrics (A) and MTurk (B). (C, D) Average of facial images, as submitted by survey respondents on Qualtrics (C) and MTurk (D).

Table S1. Summary statistics for 349 variables in increasing p-value order. AUC values are for prediction based on deep image features alone. AUC values are rounded to two decimal places and p-values to four – for presentation purposes.

No.	Variable	Avg.	AUC	AUC SE	AUC-2SE	AUC+2SE	p-value
1.	Race: African American/ Black	0.14	0.96	0.02	0.93	0.99	~0
2.	Gender: Male	0.23	0.95	0.02	0.92	0.98	~0
3.	Gender: Female	0.76	0.94	0.02	0.9	0.98	~0
4.	Race: Caucasian/ White	0.73	0.88	0.02	0.83	0.92	~0
5.	Browser: Safari iPhone	0.26	0.84	0.03	0.79	0.9	~0
6.	Prefers: iPhone vs. Galaxy	0.43	0.78	0.03	0.73	0.84	~0
7.	Browser: Chrome	0.65	0.79	0.03	0.73	0.85	~0
8.	Age: >50	0.17	0.83	0.04	0.74	0.91	~0
9.	Age: <=30	0.38	0.78	0.04	0.7	0.85	~0
10.	Data source: Qualtrics panel vs. MTurk	0.87	0.81	0.04	0.72	0.89	~0
11.	Uses Snapchat	0.51	0.7	0.03	0.64	0.76	~0
12.	Prefers: Beatles vs. Michael Jackson	0.47	0.65	0.03	0.59	0.71	~0
13.	Prefers: Clothing vs. tech	0.61	0.67	0.03	0.6	0.74	~0
14.	Employment: Employed/ student	0.74	0.67	0.04	0.6	0.75	~0
15.	Employment: Unemployed and not looking	0.18	0.71	0.04	0.62	0.79	~0
16.	Body fitness	0.68	0.66	0.03	0.59	0.72	~0
17.	Uses Apple music	0.32	0.67	0.04	0.59	0.74	~0
18.	Spends 4 hours or more a day on social media	0.39	0.62	0.03	0.56	0.68	~0
19.	Lifestyle: Would do better than average in a fist fight	0.52	0.62	0.03	0.56	0.68	~0
20.	Education achieved: High school or less	0.69	0.64	0.04	0.57	0.71	0.0001
21.	Tracks news daily	0.55	0.61	0.03	0.55	0.67	0.0001
22.	Religious background: No particular religion	0.32	0.63	0.03	0.56	0.69	0.0001
23.	Food habits, attitudes: Does not have to worry about how he eats	0.39	0.61	0.03	0.55	0.67	0.0001
24.	Regularly felt emotions: Stress	0.42	0.61	0.03	0.55	0.67	0.0001
25.	Uses Instagram	0.66	0.63	0.04	0.56	0.71	0.0002
26.	Lifestyle: Would want to be a professional football player	0.2	0.66	0.05	0.57	0.76	0.0002
27.	Uses Tinder	0.16	0.65	0.04	0.56	0.73	0.0002
28.	Uses Whatsapp	0.22	0.63	0.04	0.55	0.71	0.0003
29.	Big 5 variable: Starts quarrels with others (reverse)	0.37	0.61	0.03	0.54	0.67	0.0004
30.	Uses Netflix	0.78	0.64	0.04	0.55	0.72	0.0004
31.	Ethics: What right does your friend have to expect you to lie in court to protect him?	0.45	0.61	0.03	0.54	0.68	0.0006
32.	Likelihood of recommending Netflix to a friend	0.77	0.63	0.04	0.55	0.71	0.0007
33.	Perception of bias: FoxNews	0.42	0.61	0.03	0.54	0.68	0.0008
34.	Considers himself religious	0.5	0.6	0.03	0.54	0.66	0.0008
35.	Age: (30; 50]	0.45	0.63	0.04	0.55	0.71	0.0008
36.	Lifestyle: Likes hiking	0.6	0.6	0.03	0.54	0.67	0.0008
37.	Prefers: Trump vs. Hillary	0.39	0.61	0.03	0.54	0.67	0.0009
38.	Big 5 personality: Neuroticism	0.26	0.6	0.03	0.54	0.67	0.001
39.	Tends to watch more than 3 hours of Netflix at a time	0.53	0.59	0.03	0.53	0.64	0.0011
40.	Lifestyle: Enjoys shopping	0.82	0.65	0.05	0.55	0.74	0.0012
41.	Big 5 variable: Gets nervous easily	0.69	0.6	0.03	0.53	0.66	0.0012
42.	Perception of bias: Huffpost	0.29	0.6	0.03	0.54	0.67	0.0013
43.	Education achieved: Undergraduate degree	0.22	0.61	0.04	0.54	0.68	0.0014
44.	Price sensitivity: Sneakers	0.87	0.65	0.05	0.55	0.74	0.0016
45.	Sports programming hours watched per week: >8	0.15	0.63	0.05	0.54	0.72	0.0017
46.	Household income: <$50K	0.61	0.6	0.03	0.53	0.66	0.002
47.	Frequency of entertaining others at home	0.53	0.59	0.03	0.52	0.65	0.0027
48.	Watches Netflix 4 or more days per week	0.5	0.59	0.03	0.52	0.65	0.0029
49.	Prefers: Capitalism vs. socialism	0.38	0.59	0.03	0.52	0.65	0.0029
50.	Political party alignment: Republican	0.25	0.6	0.04	0.53	0.68	0.0031
51.	Prefers: Beach vs. mountain	0.68	0.59	0.03	0.52	0.65	0.0032
52.	Perceived effect of Superbowl ads on choices	0.33	0.59	0.03	0.53	0.66	0.0033
53.	Prefers: Night in club vs. night with a book	0.26	0.61	0.04	0.53	0.69	0.0036
54.	Uses Spotify	0.48	0.58	0.03	0.52	0.64	0.0039
55.	Race: Hispanic/ Latino	0.05	0.68	0.07	0.54	0.82	0.0039
56.	Employment: Unemployed, but looking	0.08	0.61	0.04	0.53	0.7	0.0042
57.	More important: Being beautiful vs. being smart	0.16	0.61	0.04	0.53	0.69	0.0046
58.	Lifestyle: Smokes too much	0.34	0.59	0.03	0.52	0.65	0.0046
59.	Lifestyle: Buys new things before others	0.47	0.57	0.03	0.52	0.63	0.0049
60.	Big 5 personality: Agreeableness	0.63	0.59	0.04	0.52	0.67	0.0051
61.	Food habits, attitudes: Is satisfied with his weight	0.5	0.58	0.03	0.52	0.65	0.0054
62.	Race: Asian	0.02	0.75	0.1	0.55	0.95	0.0055
63.	Political party alignment: Democrat	0.46	0.59	0.04	0.52	0.67	0.006
64.	Uses Github	0.1	0.63	0.05	0.53	0.74	0.0064
65.	Perception of bias: Breitbart	0.27	0.6	0.04	0.52	0.68	0.0065
66.	Sports programming hours watched per week: 0	0.4	0.59	0.04	0.52	0.66	0.0066
67.	Lifestyle: Likes to play poker	0.37	0.58	0.03	0.52	0.65	0.0068
68.	Perception of bias: New York Times	0.26	0.59	0.04	0.52	0.67	0.0069
69.	Big 5 variable: Has few artistic interests (reverse)	0.63	0.58	0.03	0.51	0.65	0.0076
70.	Religious background: Christianity	0.5	0.58	0.03	0.51	0.64	0.0079
71.	Sexual orientation (non-hetero)	0.15	0.6	0.04	0.52	0.68	0.0083
72.	Uses Amazon	0.61	0.57	0.03	0.51	0.64	0.0083
73.	Uses WSJ	0.24	0.58	0.04	0.51	0.66	0.0084
74.	Food habits, attitudes: Never thinks of healthy or unhealthy food	0.43	0.57	0.03	0.51	0.63	0.0085
75.	Big 5 variable: Tends to be lazy (reverse)	0.54	0.57	0.03	0.51	0.64	0.0086
76.	More important: Friendship vs. laws	0.8	0.6	0.04	0.52	0.69	0.0087
77.	Lifestyle: Likes war stories	0.47	0.57	0.03	0.51	0.63	0.009
78.	Perception of bias: Vice	0.21	0.6	0.04	0.51	0.68	0.0098
79.	Big 5 variable: Tends to be disorganized (reverse)	0.55	0.57	0.03	0.51	0.63	0.0101
80.	Big 5 variable: Tends to find faults with others (reverse)	0.58	0.57	0.03	0.51	0.64	0.0101
81.	Active consumer: Boston Globe	0.06	0.64	0.06	0.52	0.76	0.0115
82.	Lifestyle: Thinks there is too much violence on TV	0.62	0.58	0.04	0.51	0.66	0.0117
83.	Uses Yahoo	0.62	0.56	0.03	0.51	0.62	0.0119
84.	Prefers: Original coke vs. diet	0.78	0.59	0.04	0.51	0.68	0.0123
85.	Uses Telegram	0.11	0.62	0.05	0.51	0.73	0.0131
86.	Big 5 personality: Conscientiousness	0.57	0.57	0.03	0.51	0.63	0.0138
87.	Perception of bias: CNN	0.31	0.58	0.03	0.51	0.64	0.0141
88.	Big 5 variable: Is emotionally stable, not easily upset (reverse)	0.73	0.58	0.03	0.51	0.65	0.0142
89.	Regularly felt emotions: Surprise	0.21	0.58	0.04	0.51	0.66	0.0145
90.	Breakfast food choice: Potatoes	0.14	0.61	0.05	0.51	0.7	0.0146
91.	Likelihood of social media post about positive shopping experience	0.6	0.59	0.04	0.51	0.66	0.015
92.	Lifestyle: Likes danger	0.3	0.57	0.03	0.51	0.64	0.015
93.	Active consumer: Twitter	0.26	0.58	0.04	0.51	0.66	0.0152
94.	Uses Pandora	0.59	0.57	0.03	0.5	0.63	0.0156
95.	Uses NYT	0.29	0.58	0.04	0.51	0.66	0.0159
96.	More important: Family vs. career	0.88	0.61	0.05	0.51	0.72	0.0159
97.	Big 5 variable: Can be moody	0.78	0.58	0.04	0.51	0.66	0.0162

Continued from previous page

No.	Variable	Avg.	AUC	AUC SE	AUC-2SE	AUC+2SE	p-value
98.	Social class	0.45	0.57	0.03	0.5	0.64	0.0168
99.	Lifestyle: Is good at fixing mechanical things	0.48	0.59	0.04	0.5	0.67	0.0169
100.	Regularly felt emotions: Anger	0.18	0.59	0.04	0.5	0.67	0.017
101.	Breakfast food choice: Pasta	0.05	0.65	0.07	0.51	0.78	0.0176
102.	Price sensitivity: Smartphone	0.67	0.58	0.04	0.5	0.66	0.0182
103.	Prefers to watch videos: Online	0.61	0.57	0.03	0.5	0.63	0.0185
104.	Breakfast food choice motivations: Fits with who he is	0.2	0.58	0.04	0.5	0.66	0.0185
105.	Is a frequent alcohol consumer	0.37	0.57	0.03	0.5	0.64	0.0192
106.	Actively recommends movies to watch to friends	0.74	0.57	0.04	0.5	0.65	0.0195
107.	Active consumer: BBC News	0.14	0.6	0.05	0.5	0.69	0.0198
108.	Regularly felt emotions: Sadness	0.24	0.57	0.04	0.5	0.65	0.0199
109.	Big 5 variable: Can be somewhat careless (reverse)	0.55	0.56	0.03	0.5	0.63	0.0206
110.	Ethics: What right does your friend have to expect you to go easy on her restaurant in your review?	0.64	0.57	0.03	0.5	0.63	0.021
111.	Price sensitivity: Jeans	0.83	0.59	0.05	0.5	0.69	0.0211
112.	Lifestyle: Is outgoing and socially confident	0.65	0.57	0.04	0.5	0.64	0.0215
113.	Lifestyle: Spends money on himself that should be spent on family	0.32	0.58	0.04	0.5	0.65	0.0216
114.	Lifestyle: Would like his own airplane	0.43	0.55	0.02	0.5	0.6	0.0217
115.	Breakfast food choice: Salad	0.06	0.66	0.08	0.5	0.83	0.0229
116.	Prefers: Recognizable brand vs. not well-known brand	0.48	0.57	0.03	0.5	0.63	0.0231
117.	Lifestyle: Repairs his own car	0.3	0.57	0.04	0.5	0.65	0.0241
118.	Perception of bias: Yahoo News	0.22	0.58	0.04	0.5	0.66	0.0244
119.	Breakfast food choice: Chips	0.06	0.63	0.07	0.5	0.76	0.0244
120.	Perception of bias: Washington Post	0.27	0.58	0.04	0.5	0.66	0.0254
121.	Likelihood of asking a friend for a movie recommendation	0.77	0.58	0.04	0.5	0.66	0.0258
122.	Breakfast food choice: Vegetables	0.07	0.64	0.07	0.5	0.78	0.026
123.	Regularly felt emotions: Loneliness	0.28	0.57	0.04	0.5	0.64	0.0268
124.	Breakfast food choice motivations: Is good food to eat with others	0.08	0.61	0.05	0.5	0.71	0.0274
125.	Regularly felt emotions: Contempt	0.2	0.57	0.04	0.5	0.65	0.0281
126.	Big 5 variable: Can be cold, aloof (reverse)	0.57	0.56	0.03	0.5	0.63	0.0286
127.	Race: Other	0.05	0.63	0.07	0.49	0.77	0.0287
128.	Active consumer: FoxNews	0.22	0.57	0.04	0.5	0.64	0.0296
129.	Big 5 variable: Is full of energy	0.77	0.57	0.04	0.5	0.64	0.0297
130.	Supports National Rifle Association (NRA)	0.5	0.56	0.03	0.5	0.63	0.0301
131.	Uses Hulu	0.52	0.56	0.03	0.5	0.62	0.0303
132.	Uses Dropbox	0.38	0.57	0.04	0.49	0.64	0.0324
133.	Regularly felt emotions: Jealousy	0.15	0.59	0.05	0.49	0.69	0.0327
134.	Active consumer: CNN	0.24	0.57	0.04	0.49	0.66	0.035
135.	Breakfast food choice: Sandwich	0.13	0.58	0.05	0.49	0.68	0.0351
136.	Breakfast food choice: No breakfast	0.23	0.56	0.03	0.49	0.63	0.0354
137.	Big 5 variable: Is easily distracted (reverse)	0.64	0.56	0.03	0.49	0.62	0.0359
138.	Lifestyle: Believes that if given a chance men would cheat on spouses	0.53	0.55	0.03	0.49	0.61	0.037
139.	Active consumer: Vice	0.09	0.61	0.06	0.49	0.74	0.037
140.	Big 5 variable: Is depressed, blue	0.61	0.56	0.03	0.49	0.63	0.0376
141.	Lifestyle: Would like to be policeman	0.24	0.57	0.04	0.49	0.65	0.0378
142.	Education achieved: Graduate degree	0.09	0.59	0.05	0.49	0.7	0.0393
143.	Facebook is good for humanity: Yes	0.38	0.55	0.03	0.49	0.62	0.0399
144.	Political party alignment: Independent	0.29	0.56	0.03	0.49	0.63	0.0405
145.	Prefers: Coke vs. Pepsi	0.58	0.55	0.03	0.49	0.61	0.041
146.	Gender: Other	0.01	0.77	0.16	0.46	1.08	0.0413
147.	Prefers: Chocolate ice cream vs. strawberry ice cream	0.61	0.55	0.03	0.49	0.62	0.0414
148.	Browser: Other	0.09	0.6	0.06	0.48	0.71	0.0416
149.	Uses Twitter	0.57	0.56	0.03	0.49	0.62	0.0417
150.	Prefers: Children vs. no children	0.72	0.57	0.04	0.49	0.65	0.0419
151.	Lifestyle: Likes hunting	0.32	0.57	0.04	0.49	0.65	0.0419
152.	Will stop buying a brand accused of offensive advertising	0.57	0.56	0.04	0.49	0.64	0.0419
153.	Lifestyle: Tends to make compulsive purchases	0.55	0.55	0.03	0.49	0.61	0.0449
154.	Prefers to watch videos: Does not watch videos	0.05	0.64	0.08	0.48	0.8	0.0452
155.	Active consumer: New York Times	0.16	0.57	0.04	0.49	0.65	0.0458
156.	Marital status	0.48	0.56	0.03	0.49	0.63	0.046
157.	More important: Wealth vs. fame	0.91	0.59	0.05	0.48	0.7	0.0464
158.	Household income: [50K,100K)	0.28	0.55	0.03	0.49	0.62	0.0473
159.	Big 5 variable: Is sometimes rude to others (reverse)	0.49	0.56	0.03	0.49	0.62	0.0474
160.	Breakfast food choice: Eggs	0.49	0.55	0.03	0.49	0.62	0.0503
161.	Uses Saks	0.16	0.57	0.04	0.49	0.65	0.0511
162.	Makes effort to recycle	0.66	0.55	0.03	0.49	0.61	0.0514
163.	ZIP code first digit: 2, 3	0.3	0.57	0.04	0.48	0.65	0.0518
164.	Lifestyle: Likes fishing	0.62	0.55	0.03	0.49	0.62	0.0518
165.	Breakfast food choice: Fish	0.03	0.66	0.1	0.46	0.85	0.0524
166.	Active consumer: Facebook	0.62	0.55	0.03	0.49	0.62	0.0525
167.	Windfall income allocation: Gift to a loved one	0.05	0.62	0.07	0.47	0.77	0.0527
168.	Breakfast food choice: Chicken	0.08	0.61	0.07	0.47	0.75	0.053
169.	Big 5 variable: Is sophisticated in arts, music, literature	0.77	0.57	0.04	0.48	0.65	0.0536
170.	ZIP code first digit: 8, 9	0.16	0.56	0.04	0.48	0.64	0.0539
171.	Reads product review in detail before purchase	0.78	0.56	0.04	0.48	0.64	0.0539
172.	Perception of bias: Twitter	0.27	0.56	0.04	0.48	0.64	0.054
173.	Regularly felt emotions: Joy	0.46	0.55	0.03	0.49	0.61	0.0556
174.	Big 5 variable: Is outgoing, sociable	0.78	0.56	0.04	0.48	0.64	0.0558
175.	Breakfast food choice: Cold cereal	0.41	0.55	0.03	0.49	0.62	0.0559
176.	ZIP code first digit: 0, 1	0.1	0.6	0.06	0.47	0.72	0.0569
177.	Price sensitivity: Washing machine	0.72	0.55	0.04	0.48	0.63	0.0611
178.	Perception of bias: BBC News	0.19	0.57	0.04	0.48	0.65	0.0631
179.	Religious background: Judaism, Islam	0.03	0.64	0.09	0.46	0.83	0.0631
180.	Breakfast food choice: Brownies, snack, cakes	0.07	0.6	0.07	0.47	0.74	0.0646
181.	Big 5 variable: Has assertive personality	0.79	0.56	0.04	0.48	0.64	0.0652
182.	Believes offensive ads should be banned	0.57	0.55	0.04	0.48	0.63	0.0675
183.	Perception of bias: WSJ	0.23	0.56	0.04	0.48	0.63	0.0689
184.	Regularly felt emotions: Regret	0.25	0.55	0.04	0.48	0.62	0.0691
185.	Prefers: Madonna vs. Lady Gaga	0.45	0.55	0.03	0.48	0.62	0.0691
186.	Lifestyle: Is highly social with many friends	0.54	0.55	0.03	0.48	0.61	0.0712
187.	Regularly felt emotions: Happiness	0.49	0.55	0.03	0.48	0.62	0.0719
188.	Active consumer: Google news	0.29	0.56	0.04	0.48	0.63	0.0723
189.	Breakfast food choice: Frozen_waffle	0.19	0.56	0.04	0.48	0.64	0.0745
190.	Price sensitivity: Microwave	0.75	0.56	0.04	0.48	0.63	0.0749
191.	Big 5 variable: Worries a lot	0.75	0.56	0.04	0.48	0.65	0.0767
192.	Uses Shazam	0.2	0.56	0.04	0.47	0.65	0.0782
193.	Breakfast food choice: Soup	0.04	0.63	0.09	0.44	0.82	0.081
194.	Active consumer: Chicago Tribune	0.07	0.59	0.06	0.46	0.72	0.0829
195.	Household income: >=$100K	0.11	0.58	0.06	0.46	0.69	0.0884
196.	Perception of bias: Chicago Tribune	0.19	0.56	0.04	0.47	0.64	0.0906
197.	Price sensitivity: Laptop	0.89	0.57	0.05	0.47	0.67	0.0912

Continued from previous page

No.	Variable	Avg.	AUC	AUC SE	AUC-2SE	AUC+2SE	p-value
198.	Big 5 variable: Is relaxed, handles stress well (reverse)	0.75	0.55	0.04	0.47	0.62	0.0941
199.	Perception of bias: Facebook	0.35	0.54	0.03	0.48	0.61	0.0951
200.	Breakfast food choice: Bread, toast	0.36	0.54	0.03	0.48	0.61	0.0957
201.	Regularly felt emotions: Warmth	0.47	0.54	0.03	0.48	0.61	0.0985
202.	Religious background: Other (Hinduism, Buddhism, etc.)	0.16	0.56	0.05	0.47	0.65	0.0986
203.	Breakfast food choice motivations: Likes ingredients	0.21	0.55	0.04	0.47	0.63	0.1017
204.	Breakfast food choice: Popcorn	0.03	0.62	0.1	0.43	0.82	0.1026
205.	Big 5 variable: Has forgiving nature	0.89	0.56	0.04	0.47	0.65	0.1027
206.	Prefers: Telling a story vs. listening to a story	0.3	0.53	0.03	0.48	0.59	0.1046
207.	More important: Pleasure vs. duty	0.59	0.54	0.03	0.47	0.61	0.1071
208.	Is better/ worse financially than a year before	0.78	0.55	0.04	0.47	0.64	0.111
209.	Regularly felt emotions: Vulnerability	0.28	0.55	0.04	0.47	0.62	0.1116
210.	Perception of bias: Boston Globe	0.19	0.55	0.04	0.46	0.64	0.1138
211.	Food habits, attitudes:: Chooses snack foods that give vitamins and minerals	0.55	0.54	0.04	0.47	0.61	0.1149
212.	Big 5 variable: Remains calm in tense situations (reverse)	0.84	0.55	0.04	0.47	0.62	0.1161
213.	Food habits, attitudes: Does not have much interest in cooking	0.3	0.54	0.03	0.47	0.61	0.1193
214.	Food habits, attitudes: Follows regular exercise routine	0.46	0.54	0.04	0.47	0.61	0.1243
215.	Lifestyle: Likes out of doors	0.79	0.54	0.04	0.47	0.62	0.1248
216.	Uses Hotmail	0.36	0.54	0.03	0.47	0.61	0.1249
217.	Likelihood of following a movie recommendation from a friend	0.87	0.56	0.05	0.46	0.66	0.1249
218.	Uses Facebook	0.91	0.55	0.05	0.46	0.65	0.1282
219.	Regularly felt emotions: Fear	0.19	0.54	0.04	0.47	0.62	0.1287
220.	Regularly felt emotions: Guilt	0.19	0.55	0.04	0.46	0.63	0.1331
221.	Breakfast food choice: Fruit	0.35	0.54	0.04	0.47	0.62	0.134
222.	Big 5 variable: Tends to be quiet (reverse)	0.76	0.54	0.04	0.46	0.62	0.1392
223.	More important: Belonging vs. independence	0.37	0.54	0.03	0.47	0.6	0.1396
224.	Lifestyle: Likes to be in charge	0.71	0.54	0.04	0.47	0.61	0.141
225.	Active consumer: Yahoo news	0.21	0.55	0.05	0.46	0.64	0.144
226.	Big 5 variable: Generates enthusiasm	0.86	0.54	0.04	0.46	0.63	0.1448
227.	Food habits, attitudes: Believes lower priced products are the same as higher priced ones	0.64	0.53	0.03	0.47	0.6	0.1464
228.	Lifestyle: Cabin by a quiet lake is a good way to spend summer	0.86	0.55	0.05	0.45	0.65	0.151
229.	Is ready to pay more for organic food products	0.39	0.54	0.04	0.47	0.61	0.1518
230.	Uses Bloomingdale's	0.17	0.54	0.04	0.46	0.63	0.1528
231.	Breakfast food choice: Hot cereal or oatmeal	0.32	0.54	0.03	0.47	0.6	0.1538
232.	Price sensitivity: Bicycle	0.84	0.55	0.05	0.46	0.64	0.1556
233.	Breakfast food choice motivations: Helps relax, reduce stress	0.12	0.55	0.05	0.45	0.66	0.1557
234.	Big 5 variable: Is sometimes shy, inhibited (reverse)	0.77	0.54	0.04	0.46	0.63	0.1576
235.	Food habits, attitudes: Seeks out healthy foods	0.68	0.53	0.03	0.47	0.6	0.16
236.	Breakfast food choice: Bagel, roll	0.28	0.53	0.03	0.47	0.6	0.1607
237.	Facebook is good for humanity: Unsure	0.35	0.53	0.03	0.47	0.6	0.1618
238.	Lifestyle: Likes to try new things	0.86	0.55	0.05	0.45	0.66	0.1631
239.	Breakfast food choice: Crackers	0.06	0.57	0.08	0.42	0.73	0.1671
240.	Price sensitivity: Office chair	0.77	0.54	0.04	0.46	0.62	0.1687
241.	Breakfast food choice motivations: Has smooth, creamy texture	0.1	0.55	0.06	0.44	0.67	0.1704
242.	Intent to get Netflix subscription within 6 months	0.07	0.55	0.05	0.44	0.66	0.1738
243.	Breakfast food choice: Pudding, gelatin	0.04	0.59	0.09	0.4	0.77	0.1755
244.	Trusts Internet news	0.51	0.53	0.03	0.46	0.6	0.1775
245.	Breakfast food choice motivations: Has refreshing taste	0.17	0.54	0.04	0.46	0.62	0.1793
246.	Big 5 variable: Is talkative	0.78	0.54	0.04	0.45	0.63	0.1794
247.	Regularly felt emotions: Disgust	0.16	0.54	0.04	0.45	0.63	0.1798
248.	Big 5 variable: Prefers routine work (reverse)	0.8	0.53	0.04	0.46	0.61	0.1803
249.	Big 5 variable: Can be tense	0.77	0.53	0.04	0.46	0.61	0.1807
250.	ZIP code first digit: 4, 5	0.2	0.54	0.04	0.46	0.62	0.1809
251.	Breakfast food choice: Pastry, buns, fruit pies	0.15	0.53	0.04	0.46	0.61	0.1821
252.	Food habits, attitudes: Looks for new products when at grocery store	0.69	0.53	0.03	0.47	0.59	0.1834
253.	Likelihood of social media post about negative shopping experience	0.48	0.53	0.03	0.47	0.59	0.1842
254.	Big 5 variable: Is reliable worker	0.96	0.58	0.09	0.41	0.75	0.1851
255.	Prefers: Thinking vs. acting	0.73	0.53	0.03	0.46	0.6	0.1884
256.	Breakfast food choice motivations: Takes care of hunger, is filling	0.35	0.53	0.03	0.46	0.59	0.1907
257.	Expects economic depression in the next five years	0.79	0.53	0.04	0.46	0.61	0.1916
258.	Windfall income allocation: Savings, emergencies	0.15	0.54	0.04	0.45	0.62	0.1968
259.	Prefers to watch videos: TV	0.34	0.53	0.04	0.46	0.6	0.2017
260.	Breakfast food choice: Chocolate candy	0.06	0.56	0.07	0.42	0.7	0.2021
261.	Breakfast food choice motivations: Helps control weight	0.17	0.53	0.04	0.46	0.6	0.208
262.	Active consumer: Washington Post	0.13	0.54	0.05	0.44	0.65	0.2098
263.	Breakfast food choice motivations: Keeps him going	0.27	0.53	0.04	0.45	0.61	0.2122
264.	Active consumer: Breitbart	0.06	0.57	0.09	0.4	0.74	0.2153
265.	Missed a credit card payment within last year	0.26	0.53	0.03	0.46	0.6	0.2163
266.	Big 5 variable: Is inventive	0.86	0.53	0.04	0.45	0.61	0.2164
267.	Food habits, attitudes: Is daring, adventurous in trying new foods	0.66	0.53	0.03	0.46	0.59	0.218
268.	Perception of bias: Google News	0.19	0.53	0.04	0.45	0.62	0.2186
269.	Frequency of posting on social media	0.7	0.53	0.04	0.45	0.61	0.2208
270.	Uses Gmail	0.93	0.55	0.07	0.42	0.68	0.2223
271.	Lifestyle: Is likely to participate in a political protest	0.47	0.53	0.03	0.46	0.6	0.2249
272.	Big 5 personality: Openness	0.6	0.52	0.03	0.46	0.58	0.2267
273.	Expects good/ bad times financially in the US within a year	0.84	0.53	0.04	0.45	0.62	0.2286
274.	Lifestyle: Is likely to donate to a beggar	0.63	0.53	0.04	0.46	0.6	0.2341
275.	Facebook is good for humanity: No	0.27	0.53	0.04	0.45	0.6	0.2363
276.	Prefers: Planning vs. spontaneity	0.65	0.52	0.03	0.46	0.58	0.2412
277.	Breakfast food choice motivations: Has great texture	0.12	0.53	0.05	0.44	0.63	0.2517
278.	Big 5 personality: Extraversion	0.28	0.53	0.04	0.45	0.6	0.2581
279.	Big 5 variable: Is considerate, kind to almost everyone	0.96	0.56	0.1	0.37	0.75	0.2617
280.	Breakfast food choice motivations: Tastes tangy, savory	0.08	0.55	0.07	0.4	0.69	0.2627
281.	Breakfast food choice motivations: Gives energy	0.34	0.52	0.04	0.45	0.59	0.2631
282.	Big 5 variable: Is generally trusting	0.89	0.53	0.05	0.43	0.64	0.2641
283.	Breakfast food choice motivations: Is not too filling	0.15	0.53	0.04	0.44	0.62	0.2645
284.	Big 5 variable: Is original, comes up new ideas	0.9	0.54	0.06	0.42	0.65	0.2678
285.	Food habits, attitudes: Grew up eating healthy foods	0.56	0.52	0.04	0.45	0.6	0.2678
286.	Regularly felt emotions: Curiosity	0.5	0.52	0.03	0.45	0.59	0.2707
287.	Sports programming hours watched per week: (0,8]	0.45	0.52	0.03	0.45	0.59	0.2719
288.	Regularly felt emotions: Optimism	0.47	0.52	0.04	0.45	0.6	0.2723
289.	Breakfast food choice motivations: Satisfies a craving	0.28	0.52	0.04	0.45	0.6	0.2746
290.	Active consumer: Huffpost	0.12	0.53	0.05	0.43	0.63	0.2753
291.	Breakfast food choice: Meat	0.16	0.53	0.05	0.43	0.63	0.2763
292.	Breakfast food choice motivations: Gives protein	0.29	0.52	0.04	0.45	0.6	0.2769
293.	Breakfast food choice: Ice cream, sorbet	0.03	0.55	0.09	0.37	0.73	0.2814
294.	Food habits, attitudes: Reads ingredient list on the label	0.68	0.52	0.03	0.46	0.58	0.2863
295.	Windfall income allocation: Necessities, bills	0.23	0.52	0.04	0.44	0.6	0.2883
296.	Breakfast food choice: Cake, cookies	0.08	0.53	0.06	0.41	0.66	0.29

Continued from previous page

No.	Variable	Avg.	AUC	AUC SE	AUC-2SE	AUC+2SE	p-value
297.	Food habits, attitudes: Finds it hard to be disciplined about what he eats	0.67	0.52	0.04	0.44	0.6	0.2932
298.	Believes global warming is a threat	0.72	0.52	0.04	0.45	0.59	0.2964
299.	Active consumer: WSJ	0.11	0.52	0.05	0.43	0.61	0.2998
300.	Regularly felt emotions: Gratitude	0.55	0.52	0.03	0.45	0.58	0.3029
301.	Lifestyle: Is satisfied with life	0.65	0.52	0.03	0.45	0.59	0.3058
302.	Big 5 variable: Is helpful, unselfish	0.93	0.54	0.07	0.39	0.68	0.3072
303.	Breakfast food choice motivations: Tides him over until next meal	0.28	0.52	0.03	0.45	0.58	0.3077
304.	Lifestyle: Believes there should be a gun in every home	0.5	0.52	0.03	0.45	0.58	0.3206
305.	Breakfast food choice: Muffin or croissant	0.25	0.52	0.04	0.44	0.59	0.3251
306.	Big 5 variable: Makes plans and follows through	0.9	0.52	0.05	0.43	0.61	0.3324
307.	Big 5 variable: Is ingenious, deep thinker	0.92	0.53	0.06	0.4	0.65	0.3325
308.	Lifestyle: Obeys rules	0.79	0.52	0.04	0.44	0.6	0.3379
309.	Breakfast food choice motivations: Has chunky, multidimensional texture	0.05	0.53	0.09	0.36	0.7	0.348
310.	Breakfast food choice: Regular yogurt	0.23	0.51	0.04	0.44	0.59	0.3556
311.	Breakfast food choice: Nuts	0.09	0.52	0.06	0.4	0.65	0.3571
312.	Breakfast food choice: Bar	0.25	0.51	0.04	0.43	0.6	0.363
313.	Breakfast food choice motivations: Tastes sweet	0.15	0.52	0.05	0.43	0.61	0.3645
314.	Lifestyle: Loves to eat	0.89	0.52	0.05	0.41	0.62	0.366
315.	Breakfast food choice motivations: Healthy, good, guilt free	0.24	0.51	0.04	0.44	0.58	0.3685
316.	Regularly felt emotions: Hopefulness	0.5	0.51	0.03	0.45	0.57	0.3688
317.	Big 5 variable: Is reserved (reverse)	0.81	0.51	0.04	0.43	0.6	0.374
318.	Breakfast food choice: Hummus	0.03	0.53	0.1	0.33	0.74	0.3798
319.	Food habits, attitudes: Often prepares sauces, dips from scratch	0.56	0.51	0.03	0.44	0.58	0.3812
320.	Trusts TV news	0.59	0.51	0.03	0.45	0.57	0.3819
321.	Breakfast food choice: Pretzels	0.05	0.52	0.08	0.36	0.69	0.3826
322.	Breakfast food choice motivations: Keeps him on track	0.16	0.51	0.04	0.42	0.6	0.3931
323.	Big 5 variable: Has active imagination	0.92	0.52	0.06	0.39	0.64	0.3981
324.	Breakfast food choice: Cheese, cottage cheese	0.11	0.51	0.05	0.42	0.61	0.4029
325.	Breakfast food choice motivations: Is comforting, soothing	0.19	0.51	0.04	0.43	0.59	0.4105
326.	Lifestyle: Plans spending carefully	0.72	0.51	0.03	0.45	0.57	0.412
327.	Food habits, attitudes: Sticks to healthy diet for his family	0.63	0.51	0.03	0.44	0.58	0.4145
328.	Breakfast food choice: Smoothie	0.16	0.51	0.04	0.43	0.59	0.4285
329.	Big 5 variable: Perseveres until task is finished	0.93	0.51	0.07	0.38	0.64	0.4288
330.	Food habits, attitudes: Likes ethnic foods	0.76	0.51	0.04	0.42	0.59	0.4522
331.	Big 5 variable: Is curious about many different things	0.94	0.5	0.06	0.37	0.63	0.47
332.	Regularly felt emotions: Contentness	0.37	0.5	0.04	0.43	0.58	0.4728
333.	Breakfast food choice motivations: Keeps from overeating during next meal	0.13	0.5	0.04	0.42	0.59	0.4773
334.	ZIP code first digit: 6, 7	0.23	0.5	0.03	0.43	0.57	0.483
335.	Breakfast food choice motivations: Helps maintain mental focus	0.17	0.5	0.05	0.41	0.59	0.4897
336.	Big 5 variable: Does things efficiently	0.94	0.5	0.07	0.36	0.64	0.4897
337.	Food habits, attitudes: Look for authentic ingredients and flavors	0.72	0.5	0.03	0.44	0.57	0.4907
338.	Big 5 variable: Does thorough job	0.95	0.49	0.07	0.36	0.63	0.5299
339.	Breakfast food choice: refrigerated dip (salsa, guacamole, dairy)	0.02	0.49	0.12	0.26	0.72	0.5324
340.	Big 5 variable: Likes to cooperate with others	0.93	0.49	0.07	0.35	0.63	0.5512
341.	More important: Freedom vs. truth	0.39	0.5	0.03	0.43	0.56	0.5514
342.	Lifestyle: Prefers a set routine	0.79	0.49	0.04	0.42	0.57	0.5779
343.	More important: Politeness vs. honesty	0.16	0.49	0.04	0.4	0.58	0.5825
344.	Big 5 variable: Likes to reflect, play with ideas	0.92	0.49	0.05	0.38	0.6	0.6012
345.	Big 5 variable: Values artistic, aesthetic experience	0.9	0.48	0.05	0.38	0.59	0.6226
346.	Expects to be better/ worse financially in a year	0.94	0.47	0.07	0.32	0.62	0.653
347.	Breakfast food choice motivations: Tastes great	0.39	0.49	0.04	0.42	0.56	0.6602
348.	Considers it to be a good time to buy a major household item	0.87	0.48	0.04	0.4	0.56	0.689
349.	Breakfast food choice: Greek yogurt	0.19	0.48	0.04	0.4	0.56	0.6931

Table S2. Pearson correlation of the body fitness and iPhone Safari browser variables with 30 variables that are most significantly predictable based on deep image features alone. Correlations we refer to in text are highlighted (these are all significant at two-tailed significance level 0.01 using individual-clustered standard errors).

		Pearson correlation	
**No.**	**Variable**	**Body fitness**	**Browser: Safari iPhone**
1.	Race: African American/ Black	0.08	0.04
2.	Gender: Male	0.11	-0.18
3.	Gender: Female	-0.11	0.18
4.	Race: Caucasian/ White	-0.13	-0.05
5.	Browser: Safari iPhone	0.02	1.00
6.	Prefers: iPhone vs. Galaxy	0.07	**0.60**
7.	Browser: Chrome	0.04	-0.80
8.	Age: > 50	-0.12	-0.09
9.	Age: ≤ 30	0.14	0.22
10.	Data source: Qualtrics panel vs. MTurk	-0.06	0.22
11.	Uses Snapchat	0.12	**0.20**
12.	Prefers: Beatles vs. Michael Jackson	-0.05	-0.04
13.	Prefers: Clothing vs. tech	-0.03	**0.16**
14.	Employment: Employed/ student	**0.19**	0.01
15.	Employment: Unemployed and not looking	**-0.26**	-0.02
16.	Body fitness	1.00	0.02
17.	Uses Apple music	0.11	**0.27**
18.	Spends 4 hours or more a day on social media	0.06	0.07
19.	Lifestyle: Would do better than average in a fist fight	0.12	-0.02
20.	Education achieved: High school or less	-0.10	0.01
21.	Tracks news daily	0.11	-0.12
22.	Religious background: No particular religion	0.00	-0.05
23.	Food habits, attitudes: Does not have to worry about how he eats	**0.32**	0.00
24.	Regularly felt emotions: Stress	**-0.11**	0.07
25.	Uses Instagram	0.08	0.10
26.	Lifestyle: Would want to be a professional football player	0.15	-0.04
27.	Uses Tinder	**0.14**	0.03
28.	Uses Whatsapp	0.15	-0.03
29.	Big 5 variable: Starts quarrels with others (reverse)	0.12	0.05
30.	Uses Netflix	0.04	0.08

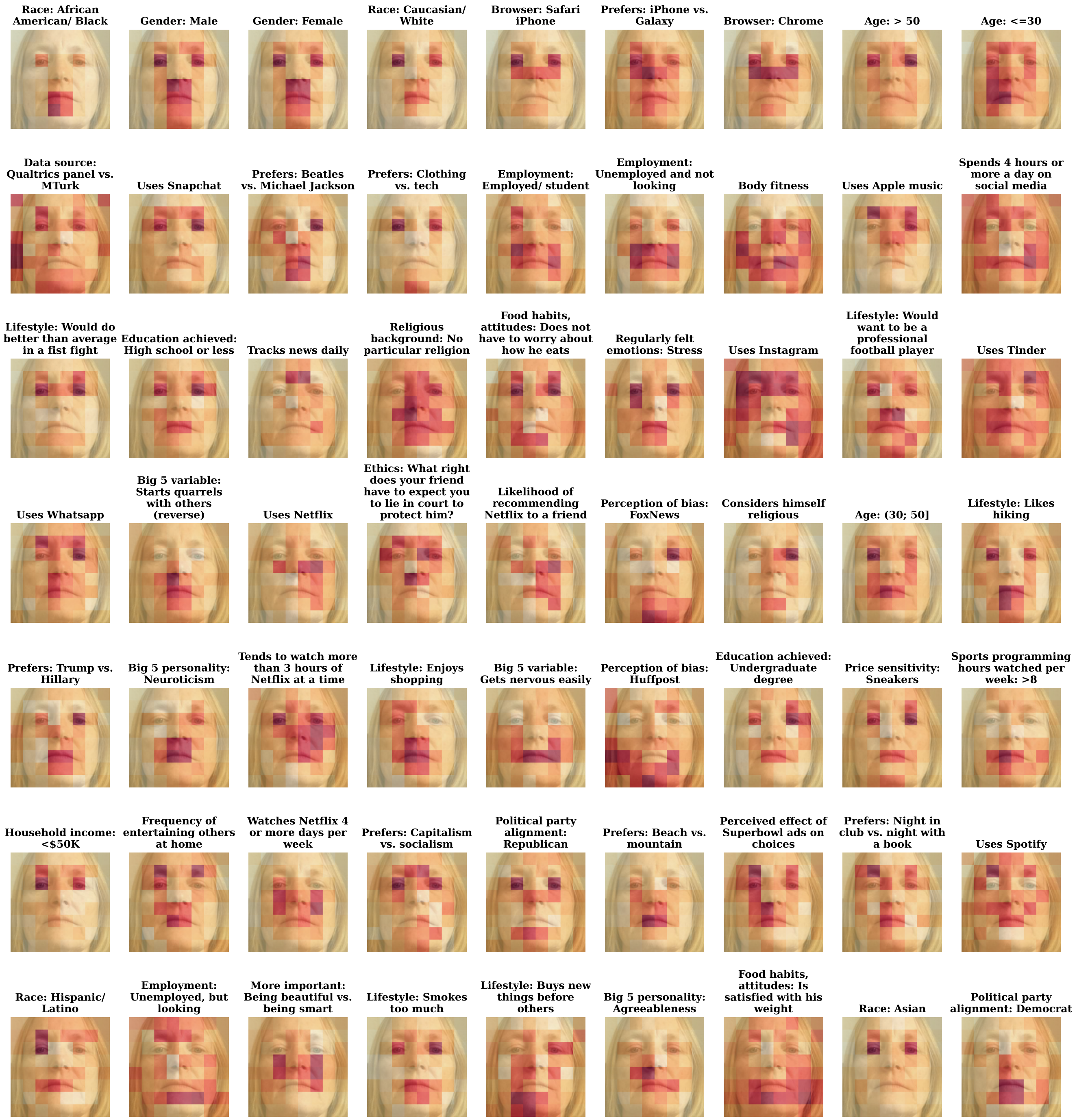

Figure S2. Importance of image segments for the quality of prediction from deep image features alone. Heat map indicates relative magnitude of AUC decrease for a variable when a square area of an image is masked (more intense color means greater decrease), avg. across images and CV folds. Background is a selected representative image. Variables are presented in the descending order of prediction significance (top 63 predictable variables are shown).

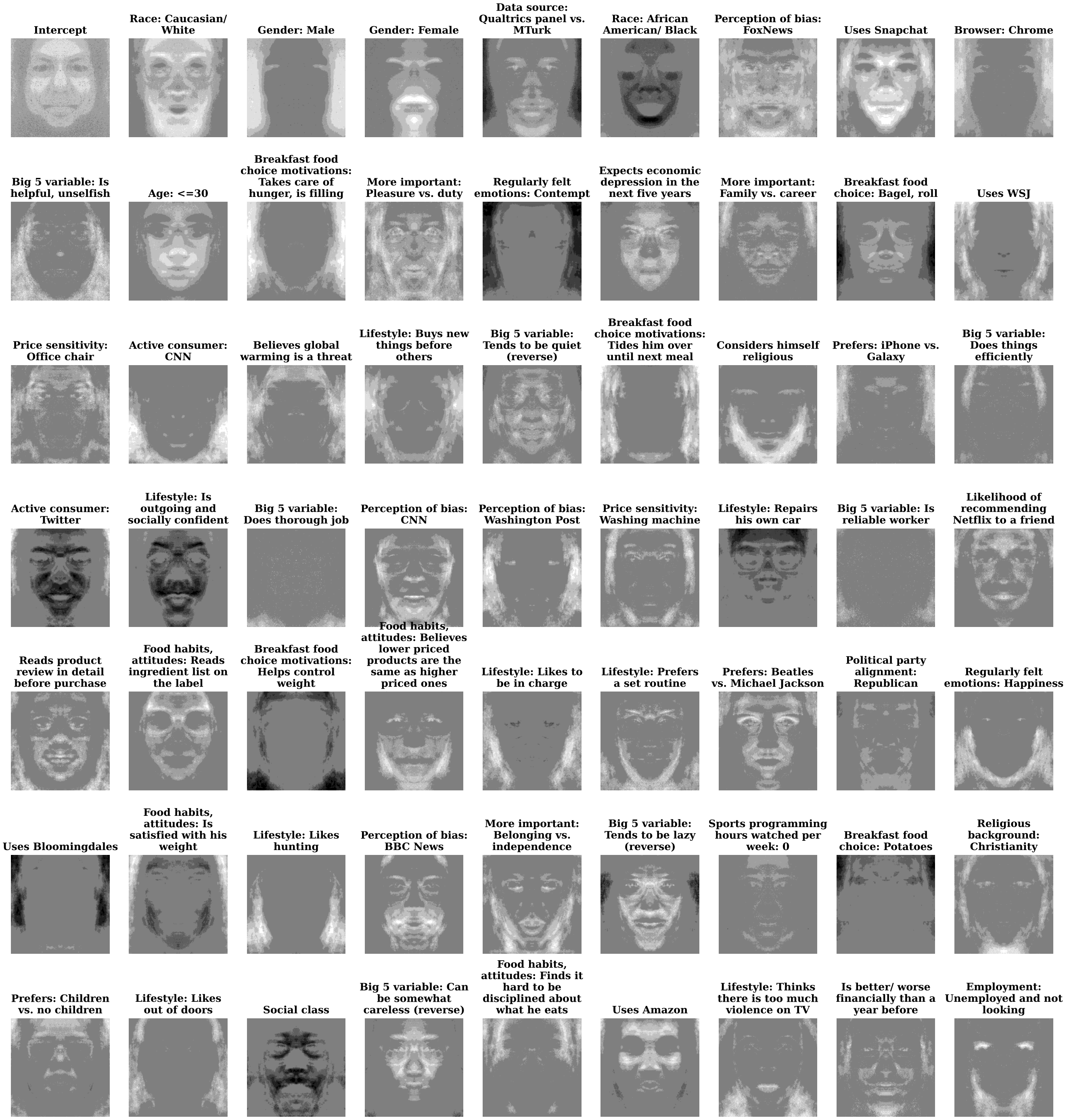

Figure S3. Coefficient matrices P_j from a regression of facial images X on binary features Y under L1 regularization. Lighter color indicates more positive coefficient values, darker color – more negative coefficient values, and gray color indicates values close to zero. Coefficients have been normalized for visualization: $P_j/\max(|P_j|)$. The coefficient matrices represent visually prominent imprinting of different features on consumer facial images, as identified by the linear model. Coefficient matrices are presented in the decreasing order of the visual prominence score $\mathbf{1}^T|P_j|\mathbf{1}$.

Data collection and pre-processing

The online questionnaire used to collect facial images and personal data was administered to respondents recruited via Qualtrics panel and MTurk. Data collection was conducted from November 2018 to February 2019. We initially started with data collection on MTurk, however, the traffic of new respondents from MTurk was insufficient in our view, so we extended data collection to Qualtrics panel participants to reach a sufficient sample size. On MTurk, we handled posting of the survey and respondent compensation ourselves directly. As to Qualtrics panel, we contracted with and provided the questionnaire link to the Qualtrics team, which handled all distribution and interactions with the survey participants. The data collection was stopped when we hit our budget constraint.

Supplementary Table S3 shows survey sample size throughout respondent dropout and data cleaning stages. The majority of dropout occurred early in the survey at the required image upload stage. Differences in dropout rates between Qualtrics panel and MTurk are likely due to different populations and recruitment mechanisms. Once a respondent submitted three photos, survey completion rate was high (87% for Qualtrics panel and 99% for MTurk).

Survey completion means a respondent gave consent to participate in the study, passed filter questions – was 18 or above and living in the US at the time of the study, uploaded three requested images (no image quality verification at this stage), and reached the end of the survey. Note that all multiple choice questions were 'force response', so respondents could not skip such questions and needed to give an answer in order to reach the survey end and thus complete the survey.

Data cleaning involved several steps. First, all surveys where respondents did not complete the survey were discarded. This ensured equal number of observations (i.e., information) per variable when training the prediction algorithm and thus the fairness of comparing predictability across variables. Second, respondents without three submitted different valid human photos were discarded. This step increased our confidence that respondents were real people with submitted photos capturing their faces. For example, respondents that submitted photos of objects, multiple identical photos, or obvious photos of celebrities were filtered out during this step. Third, respondents without a single photo surviving automated image processing (face and image feature extraction) were filtered out.

Table S3. Survey sample size by data source throughout respondent dropout and data cleaning stages

	**Qualtrics**	**MTurk**	**Total**
Respondents commencing the survey	13,327	470	13,797
+ consenting to study participation	9,198	465	9,663
+ submitting three photos (no quality verification)	1,357	126	1,483
+ completing the survey	1,187	125	1,312
+ passing manual check for three different valid photos	870	116	986
+ with at least one successfully extracted face image	853	116	969

For use in data analysis, data source (Qualtrics panel vs. MTurk) and browser type variables were extracted from survey metadata. A small number of original survey questions were not used. In particular, questions that filtered respondents out based on consent, $\geq$18 age, and US residency were not included in the analysis. Two questions asking if respondents and their parents were born in the US were also excluded as most answers to them were affirmative. Question about important factors in terms of decision to start watching Netflix was excluded due to coding issues.

Deep learning

Deep convolutional neural nets are expressive differentiable supervised learning models that have achieved state-of-the-art performance in image classification[1–3]. In this work, we use two neural nets that process images in parallel. Both neural nets use ResNet-50 deep learning architecture[1], which is known for its power and expressiveness. Our data sample is relatively small, so instead of training the model on our data from scratch, we take the approach known as transfer learning[4], where we start with a model pre-trained on a large data set. The first net, which comes with PyTorch library[5], is pre-trained on 1000-class ImageNet dataset[6], and its last several layers are fine-tuned on our data to simultaneously predict all target variables (similar to the multi-label net[7]). The fine-tuning is performed by (1) changing the last layer of the model to output predictions for the number of target variables in our data and (2) optimizing the parameters of the last 6 layers of the model via Adamax mini batch gradient descent algorithm[8] with learning rate 0.001. We train the model for 20 epochs (20 passes through all training data) with a mini batch of size 10 (each iteration, a gradient update is computed based on 10 sampled observations). When fine-tuning the deep net, we use binary cross entropy loss summed across predicted binary variables to compute the gradients. We also perform some image data augmentation. During both training and

evaluation phases, we scale image pixel values to $[0,1]$ interval and further normalize pixel values across three color channels using vectors of means $\mu = (0.485, 0.456, 0.406)$ and standard deviations $\sigma = (0.229, 0.224, 0.225)$, as required for the pre-trained ResNet-50 model distributed via PyTorch (for normalization details, see https://discuss.pytorch.org/t/what-does-it-mean-to-normalize-images-for-resnet/96160). During training only, we apply additional out of the box transformations to the facial images in order to diversify the training sample – these include (1) random horizontal flip, (2) random resize-crop operation, (3) random rotation, and (4) color jitter. We have found that fine-tuning helps boost the prediction performance of the model, compared to using unmodified pre-trained neural network to extract deep image features from images. Selection of the training setup and hyperparameters for the first neural net was performed through experimentation on UTKFace data set[9] (data downloaded from https://susanqq.github.io/UTKFace/). The second net is pre-trained for facial recognition on VGGFace2 data set[10] ('resnet50_ft' model weights downloaded from https://github.com/cydonia999/VGGFace2-pytorch) and is not fine-tuned on our data. Its use improves the quality of demographic predictions from facial images. During evaluation of the second net, we follow the same pixel scaling procedure as in case of the first net.

Analysis methodology

Repeating 5-fold cross-validation 20 times on re-shuffled data increases efficiency of the procedure (reduces the variance of holdout AUC estimates) by averaging over different possible splits of the data into folds. AUC mean and standard error across cross-validation folds for a given variable are computed as follows: $\text{Mean AUC} = \frac{1}{100}\sum_{j=1}^{100} \text{AUC}_j$; $\text{SE} = \sqrt{\frac{1}{99}\sum_{j=1}^{100}(\text{AUC}_j - \text{Mean AUC})^2}$. We could also compute the mean AUC and the SE based on 5 AUC values within each of 20 repeats and then compute average values for mean AUC and SE across repeats; we found that this alternative two-step approach yields similar results to the simpler approach that we take. AUC normality across cross-validation folds could not be rejected for ∼93% (324/349) variables, based on Shapiro-Wilk test with rejection threshold 0.05, supporting our use of Normal distribution for AUC inference.

Demographics and basic face metrics as controls in Bayesian ridge regression

Demographics are input into the Bayesian ridge regression model simultaneously as (a) individual demographic binary variables (Gender: Female, Gender: Other, Age: (30; 50], Age: > 50, Race: Asian, Race: Hispanic/ Latino, Race: African American/ Black, Race: Other; excluding redundant Gender: Male, Age: ≤ 30, Race: Caucasian/ White) and, to capture interactions, as (b) fixed effects indicating if an individual belongs to one of the 15 largest unique gender-age-race combination groups, which cover ∼93% observations in the data. Eleven basic face metrics as well as 500 deep image features (output of the SVD processing) are added directly, without interactions. The eleven basic face metrics include (1-3) RGB color channels averaged across face oval; (4) facial width-to-height ratio (fWHR) that has been connected to aggressive personality[11]; (5) face width; (6) face height; (7) nose height; (8) difference in the height of the eyes; (9) inter-eye horizontal difference; (10) mouth (lip) width; and (11) a ratio between distances from face edges to the proximate eyes.

Result robustness under different modeling choices

We have experimented with several alternative prediction pipeline configurations. Addition of VGGFace2 net as part of the deep learning model improved the quality of demographic predictions from deep image features. Use of Bayesian ridge regression instead of a ridge regression with a fixed regularization strength parameter helped circumvent the need for its explicit time-consuming cross-validation. Addition of fixed effects for unique demographic groups better encodes demographic information for its use as a control. Our core results remain directionally the same across the different modeling configurations.

References

1. He, K., Zhang, X., Ren, S. & Sun, J. Deep residual learning for image recognition. In *IEEE CVPR* (2016).
2. Benenson, R. What is the class of this image? Discover the current state of the art in objects classification. http://rodrigob.github.io/are_we_there_yet/build/classification_datasets_results.html (Accessed on Feb 23, 2020).
3. Goodfellow, I., Bengio, Y. & Courville, A. *Deep learning* (MIT Press, 2016).
4. Bengio, Y. Deep learning of representations for unsupervised and transfer learning. In *ICML*, 17–36 (2012).
5. Paszke, A. *et al.* Automatic differentiation in PyTorch. In *NIPS Autodiff Workshop* (2017).
6. Russakovsky, O. *et al.* ImageNet large scale visual recognition challenge. *International Journal of Computer Vision* **115**, 211–252 (2015).
7. Liu, L., Dzyabura, D. & Mizik, N. Visual listening in: Extracting brand image portrayed on social media. *Available at SSRN 2978805* (2019).
8. Kingma, D. P. & Ba, J. Adam: A method for stochastic optimization. *arXiv preprint arXiv:1412.6980* (2014).
9. Zhang, Z., Song, Y. & Qi, H. Age progression/regression by conditional adversarial autoencoder. In *IEEE Conference on Computer Vision and Pattern Recognition (CVPR)* (IEEE, 2017).

10. Cao, Q., Shen, L., Xie, W., Parkhi, O. M. & Zisserman, A. Vggface2: A dataset for recognising faces across pose and age. In *International Conference on Automatic Face and Gesture Recognition* (2018).

11. Carré, J. M. & McCormick, C. M. In your face: Facial metrics predict aggressive behaviour in the laboratory and in varsity and professional hockey players. *Proceedings of the Royal Society B: Biological Sciences* **275**, 2651–2656 (2008).
